# The 14-3-3σ protein promotes HCC anoikis resistance by inhibiting EGFR degradation and thereby activating the EGFR-dependent ERK1/2 signaling pathway

**DOI:** 10.7150/thno.51646

**Published:** 2021-01-01

**Authors:** Jia Song, Yachong Liu, Furong Liu, Lu Zhang, Ganxun Li, Chaoyi Yuan, Chengpeng Yu, Xun Lu, Qiumeng Liu, Xiaoping Chen, Huifang Liang, Zeyang Ding, Bixiang Zhang

**Affiliations:** Hepatic Surgery Center, Tongji Hospital, Tongji Medical College, Huazhong University of Science and Technology, Clinical Medicine Research Center for Hepatic Surgery of Hubei Province, Key Laboratory of Organ Transplantation, Ministry of Education, NHC Key Laboratory of Organ Transplantation, Key Laboratory of Organ Transplantation, Chinese Academy of Medical Sciences, Wuhan, Hubei 430030, People's Republic of China.

**Keywords:** HCC, anoikis resistance, 14-3-3σ, EGFR, ERK1/2 pathway

## Abstract

Resistance to anoikis, cell death due to matrix detachment, is acquired during tumor progression. The 14-3-3σ protein is implicated in the development of chemo- and radiation resistance, indicating a poor prognosis in multiple human cancers. However, its function in anoikis resistance and metastasis in hepatocellular carcinoma (HCC) is currently unknown.

**Methods:** Protein expression levels of 14-3-3σ were measured in paired HCC and normal tissue samples using western blot and immunohistochemical (IHC) staining. Statistical analysis was performed to evaluate the clinical correlation between 14-3-3σ expression, clinicopathological features, and overall survival. Artificial modulation of 14-3-3σ (downregulation and overexpression) was performed to explore the role of 14-3-3σ in HCC anoikis resistance and tumor metastasis* in vitro* and *in vivo*. Association of 14-3-3σ with epidermal growth factor receptor (EGFR) was assayed by co-immunoprecipitation. Effects of ectopic 14-3-3σ expression or knockdown on EGFR signaling, ligand-induced EGFR degradation and ubiquitination were examined using immunoblotting and co-immunoprecipitation, immunofluorescence staining, and flow cytometry analysis. The levels of EGFR ubiquitination, the interaction between EGFR and 14-3-3σ, and the association of EGFR with c-Cbl after EGF stimulation, in 14-3-3σ overexpressing or knockdown cells were examined to elucidate the mechanism by which 14-3-3σ inhibits EGFR degradation. Using gain-of-function or loss-of-function strategies, we further investigated the role of the EGFR signaling pathway and its downstream target machinery in 14-3-3σ-mediated anoikis resistance of HCC cells.

**Results:** We demonstrated that 14-3-3σ was upregulated in HCC tissues, whereby its overexpression was correlated with aggressive clinicopathological features and a poor prognosis. *In vitro* and *in vivo* experiments indicated that 14-3-3σ promoted anoikis resistance and metastasis of HCC cells. Mechanistically, we show that 14-3-3σ can interact with EGFR and significantly inhibit EGF-induced degradation of EGFR, stabilizing the activated receptor, and therefore prolong the activation of EGFR signaling. We demonstrated that 14-3-3σ downregulated ligand-induced EGFR degradation by inhibiting EGFR-c-Cbl association and subsequent c-Cbl-mediated EGFR ubiquitination. We further verified that activation of the ERK1/2 pathway was responsible for 14-3-3σ-mediated anoikis resistance of HCC cells. Moreover, EGFR inactivation could reverse the 14-3-3σ-mediated effects on ERK1/2 phosphorylation and anoikis resistance. Expression of 14-3-3σ and EGFR were found to be positively correlated in human HCC tissues.

**Conclusions:** Our results indicate that 14-3-3σ plays a pivotal role in the anoikis resistance and metastasis of HCC cells, presumably by inhibiting EGFR degradation and regulating the activation of the EGFR-dependent ERK1/2 pathway. To our best knowledge, this is the first report of the role of 14-3-3σ in the anoikis resistance of HCC cells, offering new research directions for the treatment of metastatic cancer by targeting 14-3-3σ.

## Introduction

Hepatocellular carcinoma (HCC) is one of the most frequently encountered malignancies in the world, and ranks as the third leading cause of cancer-related mortality 1. Despite great advances in therapeutic approaches, a high incidence of postsurgical recurrence and intrahepatic/extrahepatic metastasis are still frequently observed in the clinic, which contributes to the poor prognosis of HCC patients 2, 3. Metastasis is a crucial event in the progression of cancer and remains the main obstacle to further improving long-term survival 4. It is a complex process with multiple steps. Successful metastasis of cancer cells requires their detachment from the primary tumor, invasion through local tissue, intravasation into blood or lymph vessels, survival while in transit, extravasation, and formation of new tumors at distant sites 5. Among these prerequisite properties, anoikis resistance is considered the critical step in metastatic initiation and dissemination 6, 7. Anoikis is a special type of apoptosis that occurs when adherent cells are deprived of attachment to the extracellular matrix (ECM) 8. Acquiring anoikis resistance enables malignant cells to survive in an anchorage-independent manner and increases their survival time in circulation, as well as facilitating their eventual reattachment and colonization at secondary sites 9. Acquisition of anoikis resistance is a prerequisite for intra-hepatic spread and extra-hepatic metastasis of HCC. Therefore, understanding the mechanisms of anoikis resistance may greatly benefit the development of efficacious treatments for cancer. However, the molecular mechanisms underlying anoikis resistance in HCC are not well understood.

The 14-3-3σ protein is a member of the highly conserved family of 14-3-3 proteins, with significant roles in various cancer types 10. In colon cancer, 14-3-3σ overexpression was found to be associated with decreased survival time of patients and correlated with advanced tumor grade 11. Similarly, overexpression of 14-3-3σ was found to be associated with increased invasion and predicted a poor prognosis in pancreatic cancer 12. Moreover, an increasing number of recent studies demonstrate that increased expression of 14-3-3σ promotes tumor progression in lung adenocarcinoma 13, 14, gastric cancer 15, and cholangiocarcinoma 16. However, this protein appears to play dual roles, either promoting or inhibiting cancer progression, depending on the type of cancer. While downregulation of 14-3-3σ during cancer progression has been reported in some cancers 10, 17, 18, recent studies indicate that 14-3-3σ is upregulated in HCC 19, 20. It has been reported that expression of 14-3-3σ is associated with more aggressive tumor behavior and a poor prognosis of HCC, where it facilitates cancer progression by modulating the β-catenin/HSF-1α/HSP70 pathway 19, 20. However, the biological role and mechanism of 14-3-3σ in HCC development and tumor metastasis remain largely unknown.

The family of 14-3-3 proteins has emerged as critical regulators of anoikis in cancer. The 14-3-3ζ protein confers anoikis resistance to cancer cells by attenuating both Bad and p53 activity to suppress the intrinsic apoptotic pathway 21, 22. Liu et al. found that 14-3-3β overexpression increased anchorage-independent cell growth and promoted cancer progression in HCC 23. Similarly, previous studies found that 14-3-3σ contributes to the development of chemo- and radiation resistance, predicting a poor prognosis in multiple human cancers 24-26. Moreover, studies by Khongmanee et al., as well as Yang et al. demonstrated that expression of 14-3-3σ plays a crucial role in anoikis resistance of cholangiocarcinoma cells 16, 27. However, a direct role of 14-3-3σ in anoikis resistance and cancer metastasis has not been demonstrated in HCC.

In the present study, we show that 14-3-3σ expression was upregulated in HCC specimens. Overexpression of 14-3-3σ was found to be correlated with several aggressive clinicopathological features and disease recurrence in HCC patients. Furthermore, knockdown of 14-3-3σ reduced anoikis resistance in HCC cells and inhibited HCC metastasis *in vivo*. Our work identified a novel role for 14-3-3σ in EGFR regulation. Knockdown of 14-3-3σ dramatically reduced EGFR expression on the cell surface, accelerated EGF-induced ubiquitination and degradation of EGFR, and decreased the activation of EGFR signaling. Furthermore, we found that 14-3-3σ inhibited EGF-induced EGFR degradation after EGFR activation by interfering with EGFR-c-Cbl association and subsequent c-Cbl mediated EGFR ubiquitination. Significantly, we demonstrate that the regulatory effect of 14-3-3σ on the EGFR-ERK1/2 pathway is the key mechanism by which 14-3-3σ promotes anoikis resistance in HCC cells.

## Materials and Methods

### Patients and HCC tissue specimens

Two independent cohorts involving 133 HCC patients were enrolled in this study. In cohort 1, fresh HCC samples and adjacent non-tumor tissues were collected from 48 patients who performed hepatic resection from 2012 to 2014 at the Hepatic Surgery Center, Tongji Hospital of Huazhong University of Science and Technology (HUST) (Wuhan, China). Tissues were immediately snap-frozen in liquid nitrogen and stored at -80 °C after surgical resection. Matched fresh human hepatoma and peripheral non-tumor tissues were lysed separately for western blot detection. In cohort 2, formalin-fixed, and paraffin-embedded HCC samples were obtained from 85 patients diagnosed as HCC between January 2011 and December 2013 at the same hospital. The detailed clinical data of patients in cohort 2 were described in Table 1. Ethical approval was obtained from the Ethical Committee of Tongji Hospital, Huazhong University of Science and Technology (HUST). All patients provided written informed consent for use of their tissue specimens. The study methodologies conformed to be standards set by the Declaration of Helsinki.

### Cell lines and culture conditions

Huh7, HepG2 and HEK 293 cells were purchased from China Center for Type Culture Collection (CCTCC, Wuhan, China). Hep3b, PLC/PRF, SK-hep1, HLF, MHCC-97H, and LM3 cells were obtained from the Hepatic Surgery Center, Tongji Hospital, Huazhong University of Science and Technology, Wuhan, China. The cell lines were maintained in Dulbecco's modified Eagle medium (DMEM) supplemented with 10% FBS, 100 U/mL penicillin, and 0.1 mg/mL streptomycin in a 5% CO_2_ incubator at 37 °C.

### Plasmid constructs and transfection experiments

To establish 14-3-3σ overexpressing cell lines, the human 14-3-3σ cDNA was cloned into the pBABE-puro retroviral vector (Addgene plasmid 1764). 293FT cells were then co-transfected with pBABE-14-3-3σ, or its empty vector construct, and a packaging mix, using Lipofectamine 3000 reagent (Invitrogen) to generate retroviral particles. After 48-72 h of transfection, the medium containing the retrovirus was harvested and transduced into the target cells in the presence of polybrene (8 μg/mL). Transfected cells were selected by culturing for 2 weeks in the presence of puromycin (5 μg/mL). To generate the 14-3-3σ-knockdown cell lines, two target sequences (sh14-3-3-σ-1#, sh14-3-3-σ-2#) and one non-targeting sequence (negative control, NC) were selected and cloned into the pLKO.1 vector (pLKO.1 puro, Addgene Plasmid #8453). Transfection was performed according to standard procedures. The shRNA sequences used in this study are listed as follows: sh14-3-3σ-1# (sense 5'- CCGGGCTCTCAGTAGCCTATAAGAACTCGAGTTCTTATAGGCTACTGAGAGCTTTTTG-3'; antisense 5'- AATTCAAAAAGCTCTCAGTAGCCTATAAGAACTCGAGTTCTTATAGGCTACTGAGAGC-3') and sh14-3-3σ-2# (sense 5'- CCGGCTGCCTCTGATCGTAGGAATTCTCGAGAATTCCTACGATCAGAGGCAGTTTTTG -3'; antisense 5' - AATTCAAAAACTGCCTCTGATCGTAGGAATTCTCGAGAATTCCTACGATCAGAGGCAG-3'). For the EGF-treatment experiments, cells were starved in serum free media for 24h and then replaced with media containing EGF. For bioluminescent imaging, HLF cells were transfected with a plasmid constitutively expressing luciferase and selected by culturing in 500 ng/mL G418. siRNA and negative controls that were used with transient transfection were designed and synthesized by GenePharma (shanghai, China). Cells were transfected with siRNA using lipofectamine 3000 reagent (Invitrogen) according to the manufacturer's protocol. The siRNA sequences used to target EGFR is 5'-CUCUGGAGGAAAAGAAAGU-3'.

### Immunohistochemical staining

For 14-3-3σ and EGFR detection, the paraffin samples were cut into 4-μm slides and hydrated. Tissue sections were de-paraffinized in xylene and hydrated gradually in graded alcohol. Antigen retrieval was performed by heating in a steam cooker in 0.01 mol/l citrate buffer (pH-6.0) for 10-15 min. After cooling, slides were washed with 1 × PBS (pH-7.4) and incubated with 0.3% H_2_O_2_ solution in 1 × TBS at room temperature for 20 min to block endogenous peroxidase activity. Primary anti-14-3-3σ polyclonal antibody was added to the samples and incubated overnight at 4 °C. The slides were washed thoroughly with 1 × TBS and the antibody reaction was visualized using a fresh substrate solution containing diaminobenzidine. Immunohistochemical staining was assessed by two independent pathologists with no prior knowledge of the patient characteristics. Each specimen was assigned a score according to the intensity of positive tumor cells (score = 0, none; score = 1, weak; score = 2, intermediate; and score = 3, strong) and the extent of stained cells (0-5% = 0, 5-25% = 1, 26-50% = 2, 51-75% = 3 and 76-100% = 4). The 14-3-3σ and EGFR expression were expressed as the immunostaining score, which was calculated as the sum of the proportion and intensity of the stain. Overall scores of <6 and ≥6 were defined as being low level and high level, respectively. IHC staining was performed for measurement of cleaved-caspase-3 expression in intrahepatic tumor nodules in the slides of liver as described above.

### Anoikis assay, cell viability, caspase 3 activity assay, and annexinV/propidium idide staining

Anoikis was induced by culturing cells on ultralow attachment plates (Corning) as described by others 28. At the designated time points, the suspended cells were collected and subjected to cell viability assays by Trypan Blue Staining Cell Viability Assay Kit (Beyotime Institute of Biotechnology, Shanghai, China), apoptosis assays by Caspase 3 Activity Assay Kit (Beyotime Institute of Biotechnology), and Annexin V/PI staining on FACS (BD) according to the protocols from the respective manufacturers. Cell viability was analyzed by Trypan Blue Staining Cell Viability Assay Kit (Beyotime Institute of Biotechnology) and counted by Nexcelom Cellometer Auto X4 Cell Counter (Nexcelom Bioscience, Massachusetts, USA). All viability experiments were repeated as three independent experiments, and mean percentage of cell survival was calculated along with SD. Student's t test was used for calculating statistical significance. Caspase-3 activity was assayed using a caspase-3 activity kit (Beyotime Institute of Biotechnology) and calculated as the fold change of caspase-3 activity over that of the control. Cell apoptosis was done by Annexin V-fluorescein isothiocyanate (FITC)/propidium iodide (PI) double staining using flow cytometry analysis. Cells were incubated with Annexin V-FITC and PI for 15 minutes at room temperature in the dark. The samples were processed to the FASCcan Flow Cytometer (Becton-Dickinson, Franklin Lakes, NJ, USA).

### Immunoblotting and co-immunoprepiation (Co-IP)

Proteins were blotted on a polyvinylidene fluoride membrane (GE Healthcare, Pittsburgh, PA, USA) using a semi-dry transfer unit (Bio-Rad, San Jose, CA, USA). After blocking in Tris-buffered saline containing 5% non-fat milk and 0.1% Tween-20, the membranes were incubated with primary antibodies against different proteins overnight at 4 °C, followed by incubation with secondary antibodies at room temperature for 1 h. Immunoreactivity was visualized by ECL chemiluminescence system (Bio-Rad). For Immunoprecipitation, the cells were lysed in IP Lysis Buffer (Beyotime Institute of Biotechnology) containing protease inhibitors, precleared with protein-A/G agarose (Sigma Aldrich) and incubated with anti-14-3-3σ or anti-EGFR antibody overnight on an orbital shaker at 4 °C. The immune complex was precipitated by protein-A/G agarose, washed five times and analyzed by western blot.

### *In vitro* Migration and Invasion Assays

For migration and invasion assays, Transwell filter champers (Costar, Corning, NY) were used according to the manufacturer's instructions. Cell migration or invasion was determined by staining cells with 0.1% crystal violet. Four randomly chosen visual fields were recorded and analyzed statistically using ImageJ softerware (NIH).

### Animal experiments

All of the animal studies met the National Institutes of Health guidelines (NIH publication 86-23 revised 1985), and were approved by the Committee on the Ethics of Animal Experiments of the Tongji Medical College, HUST. Male Balb/c athymic nude mice (3-4-week-old) were housed under specific pathogen free (SPF) conditions and cared for according to the institutional guidelines on animal care. The mice were randomly divided into the indicated groups (6-10 mice/group) before inoculation and a double-blinded evaluation was performed when measuring tumor volume and number of metastatic nodules. For orthotopic implantation model, 20 μl matrigel containing 1 × 10^6^ cells were injected intrahepatically with a 27-gauge needle. At four weeks, six nude mice in each group were euthanized by anesthesia overdose and the livers were collected and photographed. Tumor sizes were evaluated. For the mouse pulmonary metastasis model, 1 × 10^6^ cells were injected into the caudal vein of BALB/C nude mice. Prior to imaging, mice were anesthetized. Metastasis was monitored using the IVIS@ Lumina II system (Caliper Life Sciences, Hopkinton, MA, USA) 10 min after intraperitoneal injection of 4.0 mg of luciferin (Gold Biotech, City, Country) in 50 µL of saline. The average relative light intensity of the chest/lung area was measured utilizing Living Image software. All the mouse groups were euthanized 8 weeks later. The lungs of each mouse were separated and then fixed for H&E staining. The average number of metastatic foci in each group was counted under a microscope.

### Immunofluorescence confocal imaging

For IF microscopy, the cells were cultured in glass coverslip-bottomed culture dishes (MatTek, Ashland, MA). After the culture medium was aspirated, the cells were rinsed with PBS twice, fixed in 4% paraformaldehyde, and permeabilized with 0.5% Triton X-100 for 15 minutes. The slides were then incubated with a primary antibody in blocking solution overnight at 4°C in a humidified chamber. The glass slides were then washed three times in PBS and incubated with Alexa Fluor 568- or Alexa Fluor 488 -conjugated second antibody for 1 h at room temperature in a humidified chamber. Finally, the cover slips were incubated with 40, 60-diamidino-2-phenylindole (Sigma-Aldrich) for 15 min, and images were obtained with a phase-contrast and confocal microscopy.

### Internalization of EGFR

Cells were grown in DMEM with 10% FBS for 14 h and then were serum starved for 24 h before treating with EGF. For the assay of internalization of EGFR, the serum-starved cells were fixed with 4% paraformaldehyde after stimulated with EGF for the indicated time points. After that, cells were used in immunofluorescence assay. For flow cytometry analysis of cell surface expression levels of EGFR, cells were trypsinized and collected after incubating with EGF at the indicated time points. After washing twice with cold PBS, cells without permeation were incubated with APC anti-human EGFR Antibody (352905, BioLegend, San Diego, CA) for 30 min. After washing, cells were immediately subjected to the flow cytometry analysis.

### *In vivo* ubiquitination assay

For ubiquitination assay of EGFR, cells were transfected with i shRNA expression vector or overexpression vector as indicated. Twenty-four hour later, cells were treated with MG132 (final concentration 20 μM) for 8 h to block proteasomal degradation. Cells were lysed in IP lysis buffer and immunoprecipitated using anti-EGFR antibody followed by immunoblotting with an anti-ubiquitin antibody.

### Chemicals and antibodies

Puromycin and G-418 were purchased from Cayman Chemical (Ann Arbor, Michigan, USA). AG1478, and U0126 were purchased from Selleck. The protein A/G agarose and EGF were purchased from Sigma (St louis, MO, USA). The following antibodies were used: anti-β-actin (#8457), anti-cleaved-caspase-3 (#9664), ani-caspase-3 (#9662), anti-cleaved PARP (#5625), anti-EGFR (#4267), anti-Erk1/2 (#4695), anti-phospho-Erk1/2 (#4370), anti-p38 (#8690), anti-phospho-p38 (#9211), anti-AKT(#9272), anti-phospho-AKT (Ser473) (#4060), anti-phospho-JNK (Thr183/Tyr185) (#9251), anti-JNK (#9252), anti-HA-Tag (#3724), anti-c-Cbl (#2747), anti-ubiquitin (P4D1) (#3936) from Cell Signaling Technology (Beverly, MA, USA); anti-Flag-Tag from Sigma-Aldrich (St Louis, MO, USA), anti-GAPDH (AC002), anti-phospho-EGFR (Y1068) (AP0820) from Abclonal Technology(Shanghai, China); anti-14-3-3σ (ab14123), anti-Bim (ab7888) from Abcam (Cambridge, MA, USA); anti-Bcl-2 (12789-1-AP) from proteintech (Chicago, IL, USA); Alexa Flour 488-conjugated anti- rabbit IgG, Alexa Flour 555-conjugated anti-rabbit IgG and Alexa Flour 555-conjugated anti-mouse IgG from Thermo Scientific (Rockford, IL, USA). HRP conjugated anti-rabbit IgG, HRP conjugated anti-mouse IgG from Beyotime Institute of Biotechnology; APC anti-human EGFR antibody (#352905) used for cell surface immunofluorescence staining from BioLegend (San Diego, CA).

### Statistical analysis

SPSS statistical software v17.0 (SPSS Inc., Chicago, IL, USA) and GraphPad prism 5 software (GraphPad Software, Inc., La Jolla, CA, USA) were used to analyze all data for statistical significance. The Chi-Square test was applied to the examination of relationship between 14-3-3σ levels and clinicopathological characteristics. A Kaplan-Meier analysis was used to assess the differences in survival rates. Two-tailed Student's t-test was used for to determine the significance between groups. Statistical significance was set at *p < 0.05.

## Results

### Expression of 14-3-3σ is significantly upregulated in HCC and associated with aggressive clinicopathological features

**Figure 1:**
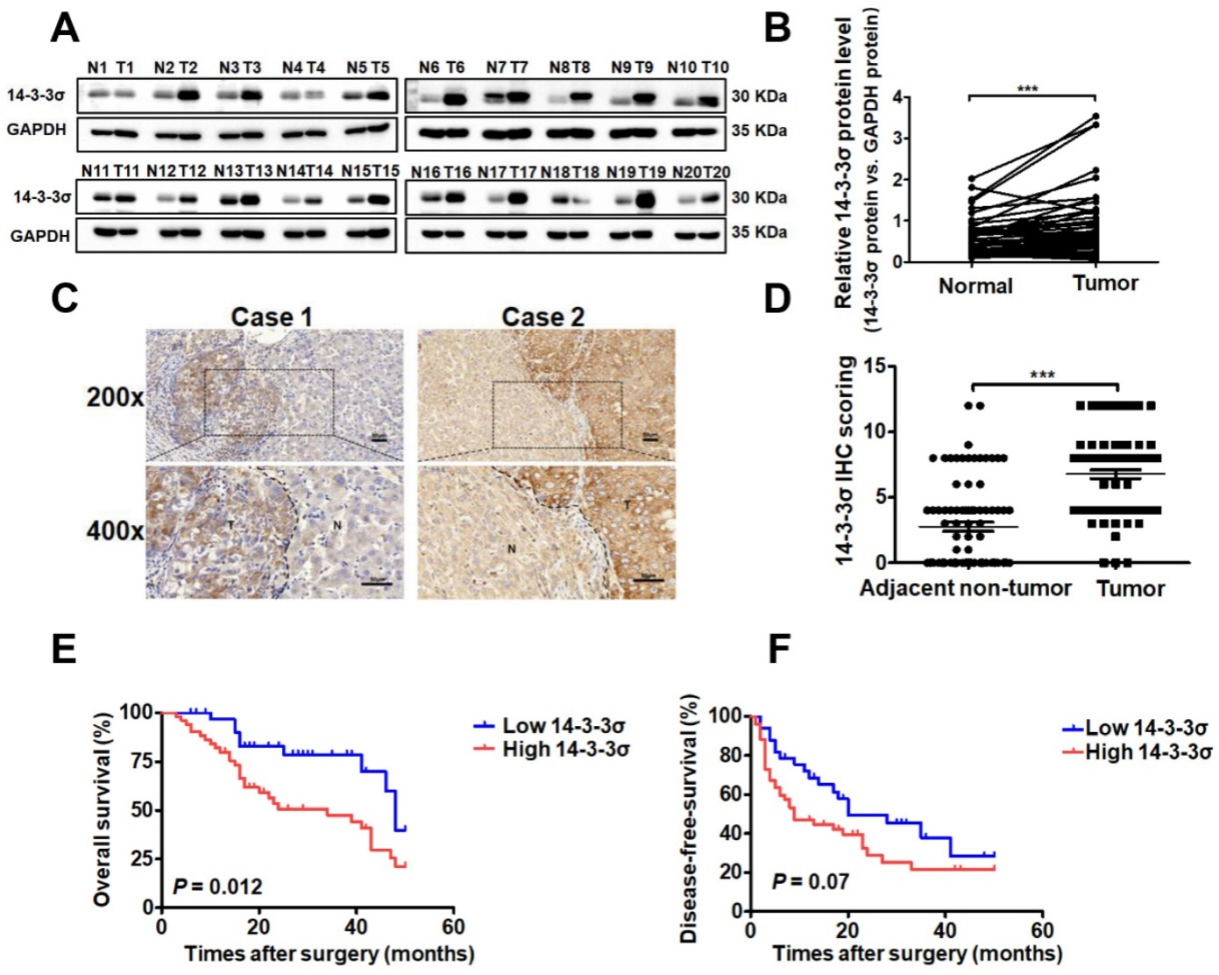
** 14-3-3σ is significantly upregulated in HCC tissues and associated with aggressive clinicopathologic features. (A)** The protein level of 14-3-3σ was analyzed in 48 paired HCCs with their corresponding non-cancerous tissues by western blot. Representative western blot results were shown. **(B)** Statistical analyses showed the upregulated level of 14-3-3σ in HCCs, as compared with that in the corresponding adjacent non-cancerous liver, where GAPDH was used as a control. ***P < 0.001. **(C)** Two representative images of immunohistochemical staining of 14-3-3σ in 85 pairs of HCC tissues were shown. Scale bars, 50 μm. **(D)** Statistical analysis of the immunohistochemistry results. ***P < 0.001. **(E)** Kaplan-Meier overall survival curve of two HCC groups: high 14-3-3σ group: n = 52; low 14-3-3σ group: n = 33. **(F)** Kaplan-Meier disease-free survival curve of two HCC groups: high 14-3-3σ group: n = 52; low 14-3-3σ group: n = 33.

To investigate the role of 14-3-3σ in the development of human HCC, we first evaluated the protein levels of 14-3-3σ in 48 paired human hepatoma and peripheral non-tumor tissue samples by western blotting. The results indicated that 14-3-3σ was significantly overexpressed in tumor tissues compared with corresponding adjacent non-tumor tissues (Figures 1A and B, and supplementary Figure S1A). To further confirm the overexpression of 14-3-3σ in HCC, the protein levels of 14-3-3σ were examined by immunohistochemical staining (IHC) in 85 pairs of HCC and adjacent normal tissues. As shown in Figures 1C and D, 14-3-3σ was significantly overexpressed in HCC specimens compared with the corresponding non-cancerous tissues (*P <* 0.001). We further investigated the associations between 14-3-3σ and the clinicopathological characteristics of patients with HCC. The chi-squared test showed that 14-3-3σ expression was correlated with several aggressive clinicopathological features, including positive microvascular invasion (*P =* 0.003), advanced TNM stage (*P =* 0.028), and advanced BCLC stage (*P* = 0.023; Table 1). Kaplan-Meier survival analysis showed that patients with high 14-3-3σ expression had poorer overall survival (OS: *P =* 0.012, Figure 1E) and disease-free survival rates (DFS: *P =* 0.027, Figure 1F) compared to patients with low 14-3-3σ expression. In addition, the online database Oncomine was used to analyze the expression of 14-3-3σ in HCC patients. As shown in Figure S1B and C, 14-3-3σ protein levels were found to be significantly upregulated in cancer tissues compared with normal tissues. We also analyzed the correlation between 14-3-3σ expression levels and clinical characteristics of HCC patients in The Cancer Genome Atlas (TCGA) database. Statistical analyses revealed that increased levels of 14-3-3σ were significantly associated with advanced clinical stage (Figure S1D), T stage (Figure S1E), M stage (Figure S1F), and residual tumor (Figure S1G). Kaplan-Meier analyses indicated that high 14-3-3σ expression was significantly correlated with poor survival of HCC patients (Figure S1H). Taken together, these data indicate that 14-3-3σ is upregulated in human HCC tissues, as well as correlated with aggressive clinicopathological features and poor prognosis of HCC patients.

### The 14-3-3σ protein enhances anoikis resistance and malignant behaviors of hepatocellular carcinoma cells

**Figure 2:**
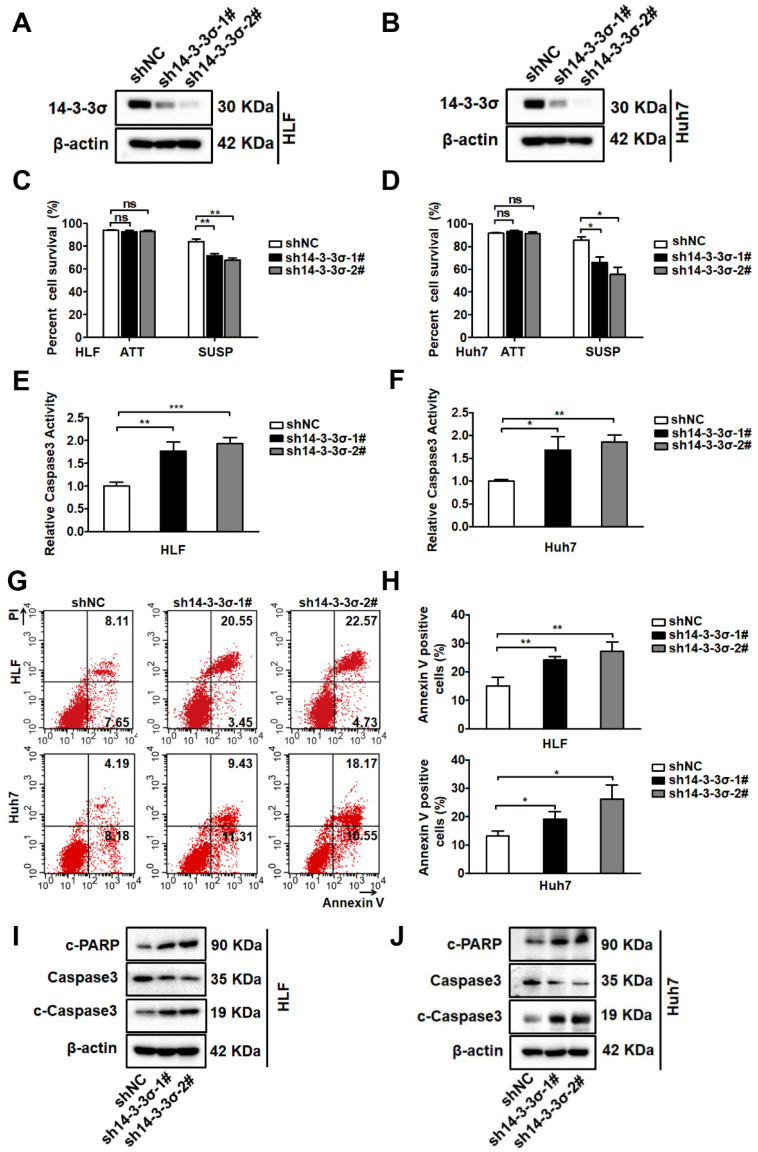
** 14-3-3σ enhances anoikis resistance in hepatocellular carcinoma cells. (A** and** B)** 14-3-3σ was stably knocked down in HCC HLF and Huh7 cells, and the protein levels of 14-3-3σ were detected by western blotting, where β-actin was used as loading control.** (C** and** D)** 14-3-3σ-knockdown HLF and Huh7 cells were grown in attached (ATT) or suspension (SUSP) conditions. 48 hours later, cell viability was analyzed by Trypan blue exclusion assay and is represented as the mean percentage cell survival of 3 independent experiments (n = 3, mean ± SD). *P < 0.05; **P < 0.01, by Student's t test.** (E** and** F)** 14-3-3σ-knockdown HLF and Huh7 cells were cultured in suspension (SUSP) conditions for 48 h. The anoikis activity was evaluated by caspase-3 activity assay and expressed relative to controls cell. Data are shown as mean ± SD (n = 3). **(G** and** H)** 14-3-3σ-knockdown HLF and Huh7 cells were cultured in suspension (SUSP) conditions. 48 hours later, cells were harvested and analyzed by flow cytometry using Annexin V kit. The representative fluorescence-activated cell sorting (FACS) analysis of HLF and Huh7 cells were shown in left **(G)** and the quantitative of Annexin V-positive cells are shown in right **(H)**. The mean value (mean ± s.d.) of three independent experiments is shown. **(I** and** J)** HLF and Huh7 cells stably transfected with shRNA against 14-3-3σ (sh14-3-3σ) and nontargeting counterpart (shNC) were cultured in suspension conditions. 48 hours later, cells were harvested and analyzed immunoblotted for the indicated proteins.

**Figure 3:**
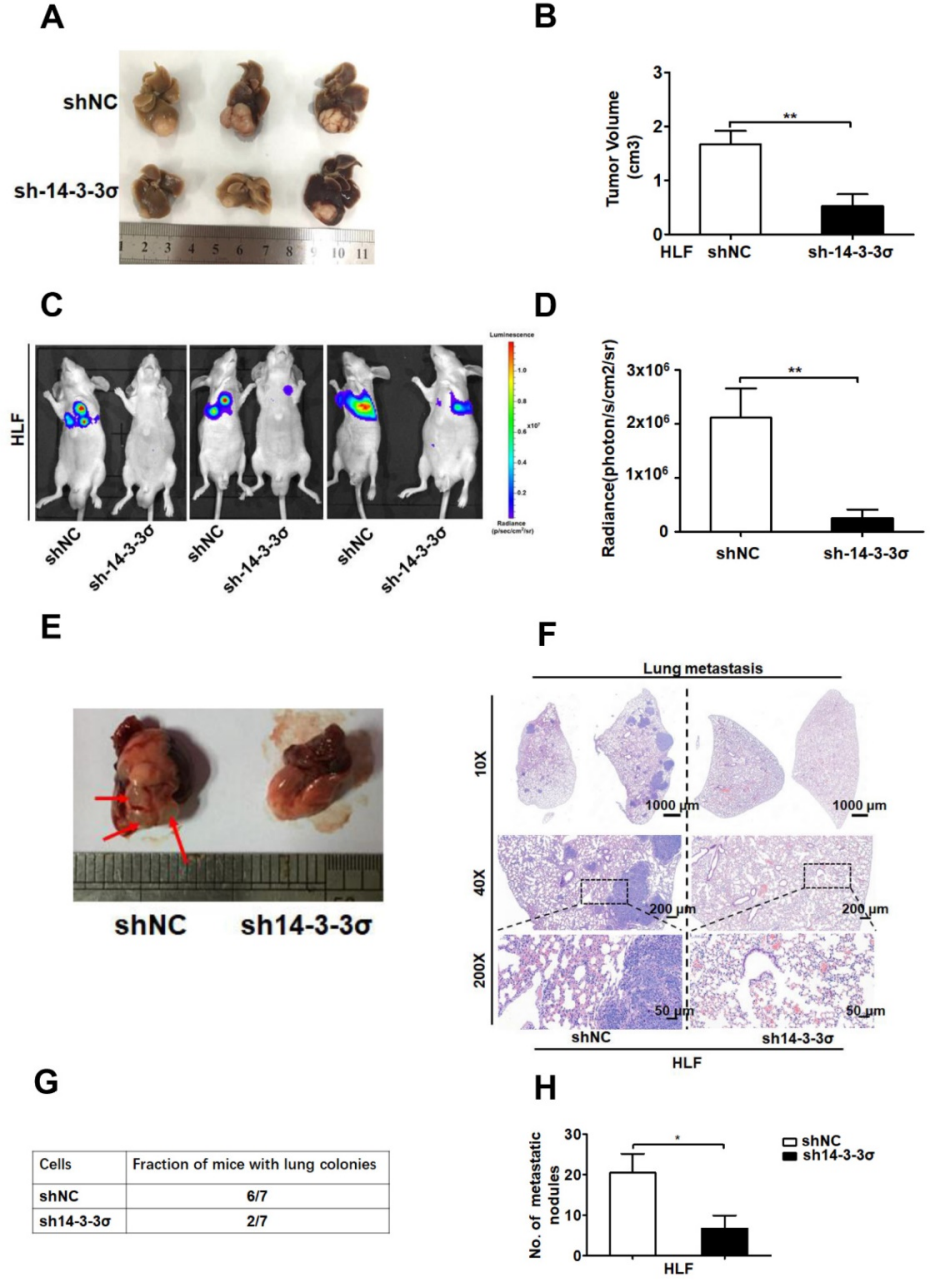
** Silencing the 14-3-3σ inhibited HCC growth and lung metastasis *in vivo*. (A)** Representative brightfield photographs of intrahepatic tumor nodules in orthotopic implantation models with indicated HLF 14-3-3σ knockdown and control cells (n = 6 for each group).** (B)** Tumor volumes of tumor foci in the orthotopic implantation models were measured and quantified. **(C)** Bioluminescent images at 8 weeks post-injection of nude mice injected intravenously with shNC (n = 7) or sh14-3-3σ (n = 7) HCC HLF cells. Representative mouse from each group is shown. **(D)** The average bioluminescent signal of the chest/lung area of mice injected intravenously with shNC or sh14-3-3σ cells was shown after injection 8 weeks. Quantification of the fluorescence signals was performed using the animal *in vivo* imaging system and its accompanying software. Data are shown as mean ± SD. **(E)** Representative massive image of lung metastases were shown. Arrow denotes visible metastatic sites.** (F)** Representative H&E staining of 2 lungs obtained from either shNC or sh14-3-3σ HCC cells injected mice. The scale bars in H&E stained tissue images are 1000 μm, 200 μm, and 50 μm, respectively. **(G)** Table summarized the incidence of lung metastases from all tail vein injection experiments with the shNC or sh14-3-3σ cells. **(H)** The histograms show the number of metastatic nodules present in the lungs from the mice (five sections were evaluated per lung). Data are expressed as means ± SD (n = 7). *P < 0.05, based on a Student's t-test.

Metastasis involves a series of sequential stages, and resistance to anoikis plays a critical role in this complicated process^5^. The biological effects of 14-3-3σ on tumor cell anoikis and the underlying mechanisms have not been clarified. To investigate if 14-3-3σ is involved in the anoikis-resistance process of HCC cells, we first compared the expression of 14-3-3σ protein between attached and suspended cells. There was a significant increase of 14-3-3σ levels in suspended cells compared with attached cells (Figures S2A and B), indicating that 14-3-3σ might play a role in the anoikis-resistance process of HCC cells. To further investigate the role of 14-3-3σ in anoikis resistance, we examined the expression of 14-3-3σ in a variety of human HCC cell lines by western blot analysis (Figure S2C). According to the 14-3-3σ levels in eight HCC cell lines, Huh7 and HLF cells were selected for silencing of 14-3-3σ, and HepG2 cells were selected for overexpression of 14-3-3σ. The efficiency of 14-3-3σ knockdown and overexpression is shown in Figures 2A, 2B, and supplementary Figure S2D. We next examined the effects of 14-3-3σ expression on anoikis resistance. We found that blocking 14-3-3σ expression during the 48-hour period following detachment significantly enhanced the anoikis rate, as characterized using the trypan blue exclusion test of cell viability (Figure 2C-D), caspase-3 activity assay (Figure 2E-F), and Annexin V/PI staining (Figure 2G-H). Like other forms of programmed cell death, anoikis is frequently characterized by the activation of caspase-3 cleavage. Moreover, to further verify that 14-3-3σ affects programmed cell death in suspension cells, the levels of caspase-3 cleavage and cleaved PARP were also analyzed by western blotting after the cells were subjected to suspension conditions. As shown in Figures 2I and J, 14-3-3σ knockdown markedly increased the levels of c-PARP and c-caspase 3 in suspension cells. We then tested whether overexpression of 14-3-3σ would cause the opposite effects. As shown in Figures S2E-H, overexpression of 14-3-3σ increased the anoikis resistance of HepG2 cells during 48h of suspension culture. Previous studies indicated that after anchorage deprival, the anoikis-resistant cells also exhibited enhanced malignant behaviors that facilitate cancer progression 29, 30. We next examined the effects of 14-3-3σ expression on cell migration and invasion. Consistently, transwell migration and matrigel invasion assays demonstrated that 14-3-3σ knockdown significantly inhibited the migration and invasion of HLF and Huh7 cells (Figures S3A and B), while overexpression of 14-3-3σ led to increased migration and invasion in HepG2 cells (Figure S3C). Taken together, these results indicated that 14-3-3σ enhanced anoikis resistance and malignant behaviors of HCC cells.

### Downregulation of 14-3-3σ suppressed HCC growth and metastasis *in vivo*

To further elucidate the functional role of 14-3-3σ in anoikis resistance *in vivo*, intrahepatic tumor implantation models with 14-3-3σ knockdown and control HLF cells were established in nude mice. As expected, the size of the HCC HLF xenografts tumors was smaller in the 14-3-3σ knockdown group compared with the control after in situ growth for 4 weeks (Figure 3A-B). Moreover, IHC analysis was performed to evaluate cleaved caspase-3 levels in tumor tissue samples derived from the HCC HLF cells. The IHC analysis demonstrated that 14-3-3σ silencing enhanced tumor cell apoptosis as revealed by cleaved caspase 3 staining, which was consistent with the *in vitro* results (Figures S4A and B). Thus, 14-3-3σ has an important role in anoikis resistance, raising the possibility that 14-3-3σ may promote tumor metastasis. To test the consequences of 14-3-3σ loss for anoikis in an *in vivo* system, we employed a lung metastasis assay using luciferase-expressing shNC or sh14-3-3σ HCC cells. The 14-3-3σ control and knockdown cells were injected into BALB/c athymic nude mice by tail vein injection to analyze the function of 14-3-3σ in distant metastasis of HCC cells.

After 8 weeks, the mice injected with the shNC HLF cells had a marked increase in luciferase activity compared with those injected with the sh14-3-3σ cells (Figures 3C and D). Then, the mice were sacrificed and the metastatic lung tumors were analyzed. Of the 7 mice injected with shNC cells, 6 developed lung colonies with increased H&E staining, compared to only 2 of the mice injected with the sh14-3-3σ cells (Figures 3E-G). At the same time, the number and size of metastatic lung tumors was significantly decreased in the nude mice injected with 14-3-3σ knockdown HLF cells compared to those injected with control cells (Figures 3F and H). Taken together, these data indicate that 14-3-3σ downregulation suppressed HCC growth and metastasis *in vivo*, indicating that 14-3-3σ plays an important role in HCC metastasis.

### The 14-3-3σ protein can interact with EGFR and modulate EGFR protein levels during EGF treatment

**Figure 4:**
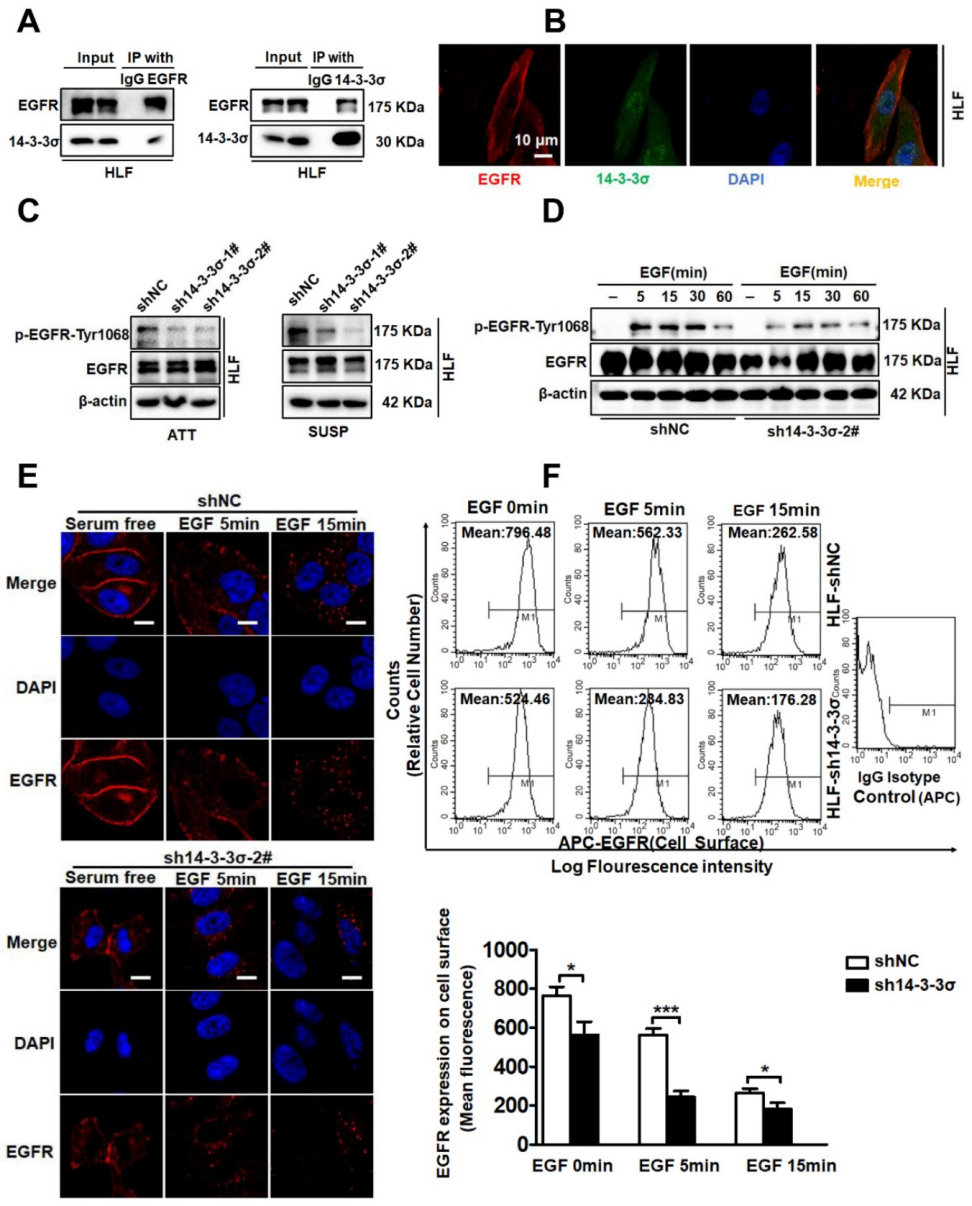
** 14-3-3σ could interact with EGFR and modulates EGFR protein levels during EGF treatment. (A)**The indicated cell lysates were prepared and subjected to co-IP with anti-EGFR or anti-14-3-3σ, followed by immunoblotting with antibody against EGFR, 14-3-3σ. **(B)** Confocal experiments were performed to determine the co-localization between endogenous EGFR and 14-3-3σ. Scale bar denotes 10 μm. **(C)** HLF shNC and sh14-3-3σ cells lines were cultured as attached (ATT) monolayers or in suspension (SUSP) conditions for 48 h. Cells were harvested and subject to western blot analysis using the indicated antibodies.** (D)** HLF shNC and sh14-3-3σ cells were serum starved overnight and treated with 40 ng/ml EGF for the indicated time period. Whole-cell lysates were prepared and subject to western blot analysis using the indicated antibodies. **(E)** HLF shNC and sh14-3-3σ cells were serum starved overnight and treated with 40 ng/ml EGF for the indicated times. Cells were fixed and analyzed by immunofluorescence confocal microscopy using an antibody against EGFR (red). Nuclei were stained with DAPI (blue). Scale bar, 10 µm. **(F)** The cell surface expression levels EGFR in HCC HLF shNC and sh14-3-3σ cells were determined using flow cytometry. The data are presented as the mean ± SD; *P < 0.05.

After confirming the involvement of 14-3-3σ in the anoikis-resistance and malignant behaviors of HCC cells, we next investigated the mechanism by which 14-3-3σ mediates anoikis resistance and metastasis. Cancer cells can achieve resistance to anoikis via a constitutive activation of pro-survival signaling, for example due to over-activation of receptors by sustained autocrine loops, oncogene activation, or growth factor receptor overexpression 8. Activation of epithelial growth factor receptor (EGFR) signaling is one of the key mechanisms through which metastatic tumor cells resist anoikis 31, 32. When cultured in suspension, HCC cells showed increased activity of EGFR signaling as determined by the levels of EGFR Thy-1068 phosphorylation, indicating that EGFR signaling is selectively activated in HCC cells to mediate anoikis resistance (Figure S5A-B). In a previous study, targeted proteomic analysis and co-immunoprecipitation was used to verify the interaction between 14-3-3σ and EGFR in nasopharyngeal carcinoma 33. Morten et al. demonstrated an association of 14-3-3 protein with the epidermal growth factor receptor (EGFR) that is rapidly induced by EGF, indicating a role of 14-3-3 proteins in EGF receptor signaling or regulation 34. Consequently, we investigated if the 14-3-3σ isoform associates with the EGFR in HCC cells. To test this, we performed reciprocal immunoprecipitation (IP) of the endogenous proteins. As expected, endogenous 14-3-3σ could associate with EGFR according to both co-immunoprecipitation (co-IP) and reverse co-IP using anti-14-3-3σ and anti-EGFR antibodies, respectively (Figure 4A). The interaction of the two endogenous proteins was further confirmed by their co-localization in HCC cells via immunofluorescence staining (Figure 4B). Given that 14-3-3σ was found to interact with EGFR, we next sought to determine whether 14-3-3σ affected the activation of the EGFR. Interestingly, the phosphorylation levels of EGFR were significantly decreased following 14-3-3σ knockdown in both attached and detached cells (Figure 4C and supplementary Figure S5C). We also investigated whether depletion of 14-3-3σ could affect EGFR phosphorylation following EGF stimulation. As shown in Figure 4D and supplementary Figure S5D, loss of 14-3-3σ impaired EGFR phosphorylation triggered by EGF in both HCC HLF and Huh7 cells compared with the corresponding control cells. The EGF/EGFR signaling pathway plays a critical role in the regulation of malignant behaviors in cancer cells, and EGF has been shown to induce the migration of HCC cells 35, 36. After demonstrating the key role of 14-3-3σ in the regulation of EGF/EGFR signaling, we also investigated whether 14-3-3σ could modulate EGF-mediated migration/invasion of HCC cells. HCC cells were treated with different concentrations of EGF, and transwell assays showed that the migration and invasion ability of HLF cells was increased by EGF stimulation, especially at 20 and 40 ng/mL (Figure S5E). Moreover, 14-3-3σ knockdown had an inhibitory effect on the EGF-induced migration and invasion activities of HLF cells (Figure S5F), while overexpression of 14-3-3σ promoted EGF-induced migration and invasion of HepG2 cells (Figure S5G). Importantly, we found that EGF-induced EGFR downregulation was significantly increased by the 14-3-3σ knockdown. To address the mechanism leading to the observed reduction of EGFR activation, we next examined EGF-induced endocytosis and degradation in response to EGF stimulation in the absence of 14-3-3σ. Confocal immunofluorescence imaging revealed that EGFR was rapidly internalized following EGF treatment in 14-3-3σ knockdown cells compared with the controls (Figure 4E and supplementary Figure S6A). In addition, these findings were also confirmed by fluorescence-activated cell sorting (FACS) analysis of EGFR levels on the surface of control and 14-3-3σ knockdown HCC cells. The 14-3-3σ knockdown cells exhibited an accelerated loss of surface EGFR signal following EGF-induced EGFR degradation compared to control cells (Figure 4F and supplementary Figure S6B). Taken together, these results indicate that 14-3-3σ significantly reduced the EGF-induced degradation of EGFR, stabilizing the activated receptor, and thereby prolonged EGFR signaling. This in turn may possibly modulate EGF-mediated malignant behaviors.

### The 14-3-3σ protein reduces EGF-induced ubiquitination of EGFR and modulates ist interaction with c-Cbl

**Figure 5:**
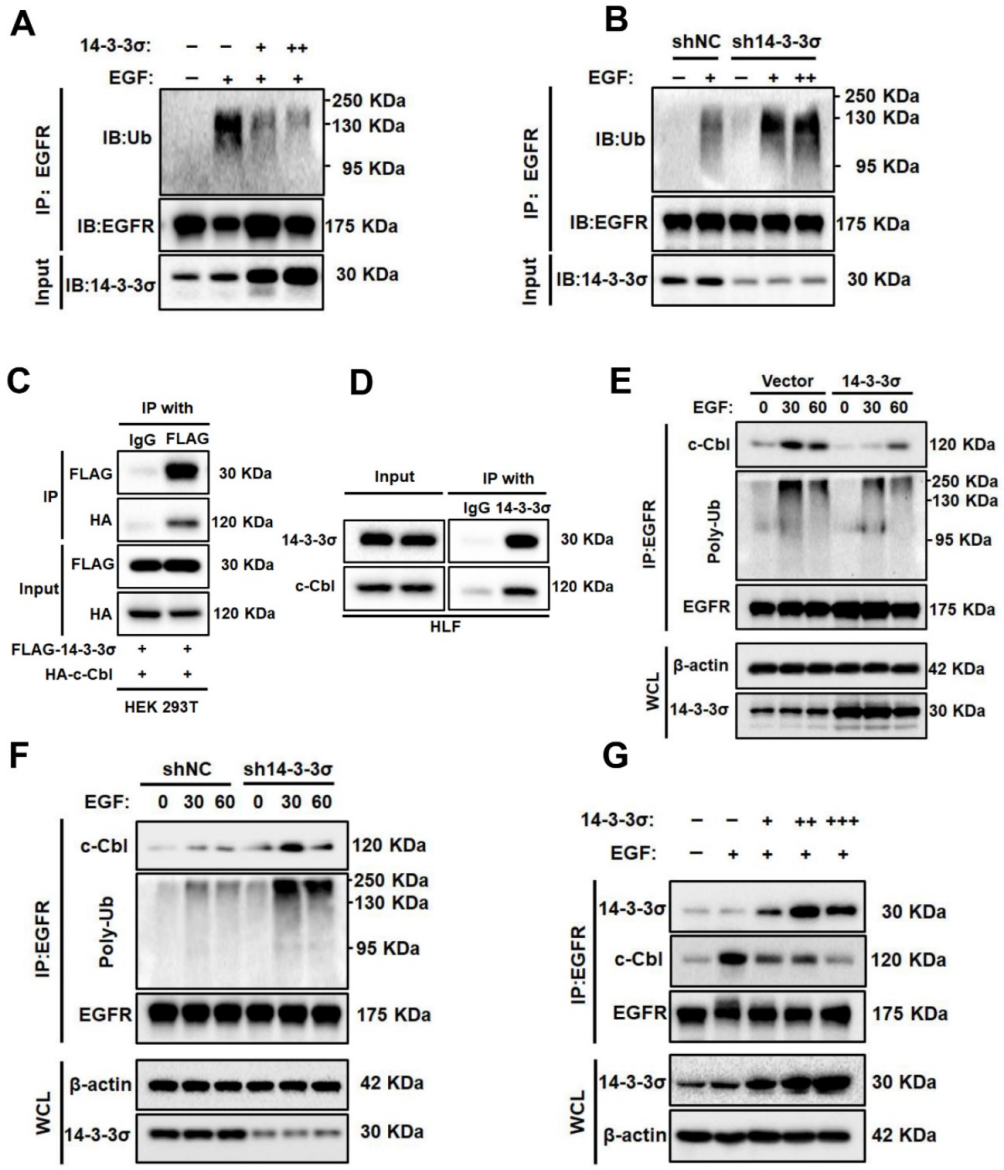
** 14-3-3σ reduces EGF-induced ubiquitination of EGFR and modulates c-Cbl-EGFR interaction.(A)** The effects of 14-3-3σ overexpression on EGFR ubiquitination: HepG2 cells were transiently transfected with 14-3-3σ-expression plasmid (+: 2 µg; ++: 4 ug) or the corresponding control vectors as indicated; then the cells were stimulated with EGF (40 ng/ml) for 15 min, and EGFR ubiquitination was analysed by immunoprecipitation and immunoblotting.** (B)** shNC or sh14-3-3σ HLF cells were starved with serum-free medium for 24 h, then were treated with the indicated concentrations of EGF (+: 40 ng/ml; ++: 80 ng/mL) for 15 min. EGFR immunoprecipitates (IP: EGFR), or whole cell lysates were subjected to Western blotting with the indicated antibodies. **(C)** Interaction between exogenous c-Cbl and 14-3-3σ. HA-c-Cbl and FALG-14-3-3σ were co-transfected into 293T cells. Cell lysates were analyzed by IP and western blotting with anti-FLAG antibodies. **(D)** Interaction of endogenous c-Cbl with 14-3-3σ determined by co-IP analyses in HLF cells. **(E)** Overexpression of 14-3-3σ inhibits EGF-induced EGFR-Cbl association and EGFR ubiquitination. HepG2 vector and 14-3-3σ overexpressing cells were serum starved overnight and treated with 40 ng/ml EGF for the indicated time period. The cells were collected and lysed, and the lysates were immunoprecipitated with anti-EGFR antibody and immunoblotted as indicated. WCL, whole-cell lysate. **(F)** Knockdown of 14-3-3σ enhances EGF induced EGFR-Cbl association and EGFR ubiquitination. shNC or sh14-3-3σ HLF cells were collected and lysed, and the lysates were immunoprecipitated and immunoblotted as described above. **(G)** HepG2 cells were transiently transfected with stepwise increasing DNA concentrations of 14-3-3σ constructs (+: 2 ug; ++: 4ug; +++: 6 ug) for 24 h. The cells were stimulated with 40 ng/ml EGF for 15 min; then the interaction between EGFR and c-Cbl, and the interaction between EGFR and 14-3-3σ were analyzed by immunoprecipitation and immunoblotting. WCL, whole-cell lysate.

Because ubiquitination plays an important role in the degradation of EGFR 37, we next examined the effect of 14-3-3σ on EGFR ubiquitination. First, the 14-3-3σ-overexpressing cells and 14-3-3σ knockdown cells were treated with EGF, after which equal amounts of EGFR were immunoprecipitated and their ubiquitination (Ub) levels were measured. The results showed that 14-3-3σ overexpression resulted inhibited the ubiquitination of EGFR in HCC Cells, while knockdown of 14-3-3σ the opposite effect (Figure 5A-B). This demonstrates that 14-3-3σ protects EGFR from EGF-induced degradation by reducing its ubiquitination. Furthermore, we investigated how 14-3-3σ controls EGFR ubiquitination. The E3-ubiquitin ligase c-Cbl has been shown to act as a switch that tightly regulates the ubiquitination of EGFR and can trigger its degradation 38. Previously, Robertson et al. identified 14-3-3σ as a binding partner of c-Cbl using the yeast two hybrid assay and GST affinity precipitation 39.

Studies have shown that interactions between 14-3-3 proteins and c-Cbl play critical roles in signal transduction processes in human neutrophils and T cells 40, 41. To clarify whether 14-3-3σ binds specifically to c-Cbl in HCC cells, we carried out co-IP and found that exogenous 14-3-3σ and c-Cbl interact in HEK293T, which was also true for the endogenous proteins in Huh7 cells (Figure 5C-D). Thus, we hypothesized that 14-3-3σ-mediated EGFR stabilization may be mediated by the downregulation of EGFR ubiquitination by c-Cbl. Consequently, we examined the effect of 14-3-3σ on EGFR/c-Cbl complex formation. The association between EGFR and Cbl was markedly reduced in 14-3-3σ-overexpressing cells compared with control cells after EGF stimulation. Consistent with the reduction of EGFR-Cbl association, the level of EGFR ubiquitination was lower in 14-3-3σ-overexpressing cells than in control cells (Figure 5E). Conversely, knockdown of 14-3-3σ in HCC cells markedly increased the levels of EGFR-Cbl association and EGFR ubiquitination (Figure 5F). At the same time, we found a correlation between the levels of EGFR-Cbl association and EGFR-14-3-3σ association in HCC cells transfected with increasing amounts of 14-3-3σ constructs. Figure 5G shows an inverse relationship, whereby the levels of EGFR-14-3-3σ association increased with the stepwise increase of 14-3-3σ expression, while the levels of EGFR-Cbl association correspondingly decreased. This suggested that 14-3-3σ may compete with Cbl for binding to the EGFR. Taken together, these results strongly indicate that 14-3-3σ can inhibit EGF-induced EGFR-Cbl association and subsequent Cbl-mediated EGFR ubiquitination.

### The 14-3-3σ protein confers anoikis resistance by activating the ERK1/2 pathway

**Figure 6:**
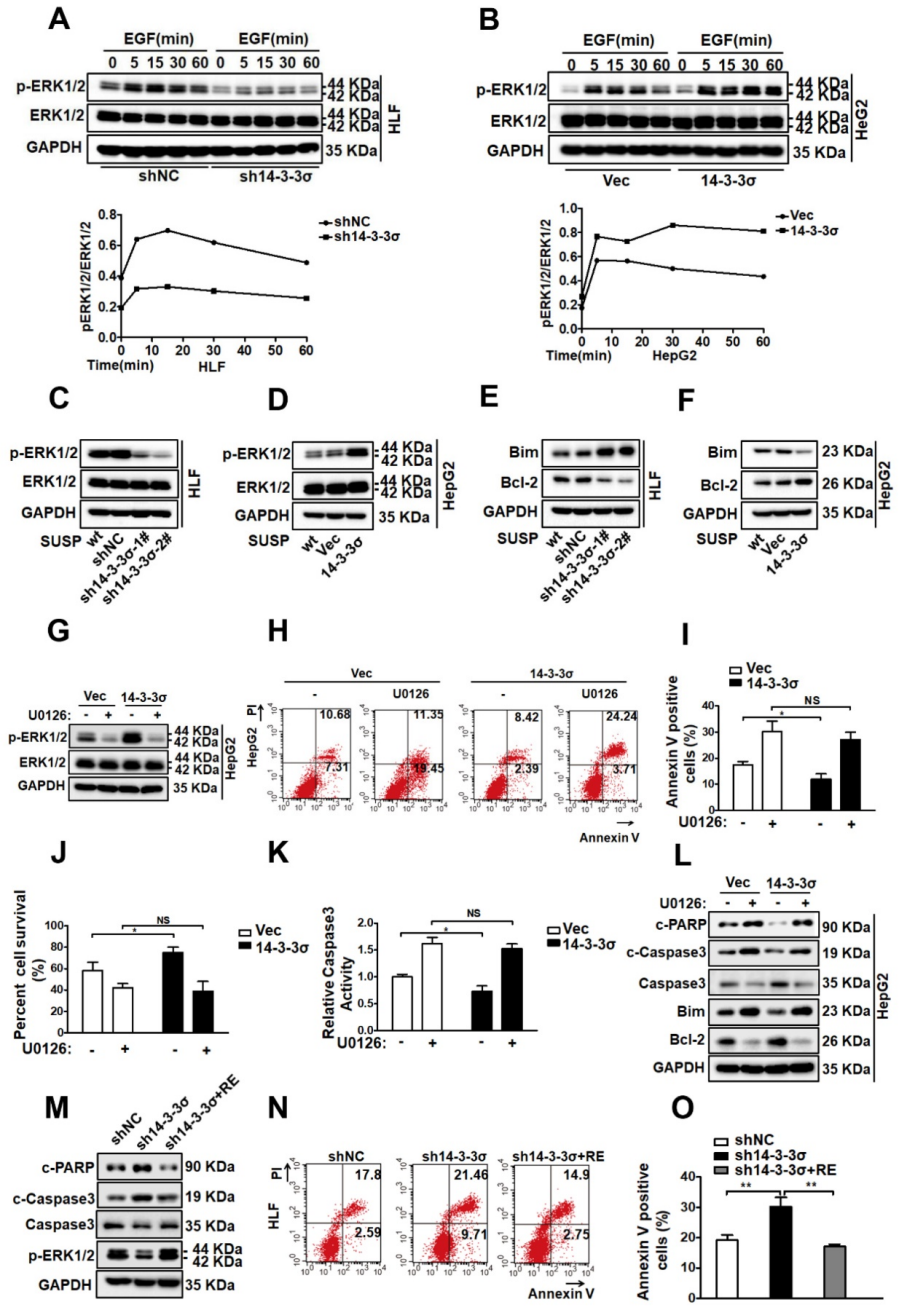
** 14-3-3σ confers anoikis resistance via the activation of the ERK1/2 pathway. (A, B)** The 14-3-3σ knock down HLF cells **(A)**, and 14-3-3σ over-expressing HepG2 cells **(B)** were starved with serum-free medium for 24 h, then added EGF (40 ng/ml) and collected at the indicated time points. The levels of ERK1/2 and phosphorylated ERK1/2 were analyzed by western blot. Densitometry graphs of p-ERK/ERK of each cell line were shown. **(C, D)** The 14-3-3σ-knockdown and 14-3-3σ over-expressing cells were grown under suspension conditions. Cell lysates were prepared 48 h after suspension culture and immunoblotted with the indicated antibodies. **(E)** Western blot analysis of Bcl2, Bim, and GAPDH for HLF cells stably transfected with shNC or sh14-3-3σ, respectively, after detachment for 48 hours. **(F)** Western blot analysis of Bcl2, Bim, and GAPDH for HepG2 cells stably overexpressing 14-3-3σ after detachment for 48 hours. **(G)** HepG2 cells with 14-3-3σ overexpression were treated with ERK1/2 inhibitor U0126 (5 μM) and cultured in suspension for 48 h. The ERK and phospho (p)-ERK1/2 protein levels were then detected by western blot analysis. **(H-L)** HepG2-Vec and HepG2-14-3-3σ cells were treated with ERK1/2 inhibitor U0126 (5 μM) and cultured in the detached state for 48 hours.** H and I**, Cell apoptosis was assessed with flow cytometric analysis using Annexin V kit. **J**, Cell viability was assessed by Trypan blue exclusion assay.** K**, cell apoptosis was assessed by caspase 3 activity assay. **L**, The expressions of Caspase-3, cleaved-caspase-3, cleaved PARP, Bim, and Bcl-2 were assessed by western blot. **(M-O)** shNC, sh14-3-3σ, and 14-3-3σ-reconstituted HLF cells were cultured in suspension for 48 h and subjected to anoikis assay as above. **M**, western blot using indicated antibodies. **N** and** O**, flow cytometric analysis using Annexin V kit.

Activation of EGFR initiates multiple common downstream signaling cascades, which include the mitogen-activated protein kinase (MAPK, also known as EKR1/2) signaling pathway, leading to cell proliferation, migration, survival, and adhesion^42^, ^43^. After confirming that 14-3-3σ is a regulator of EGFR activity in response to anoikis, and showeing that loss of 14-3-3σ resulted in decreased EGFR phosphorylation, we aimed to identify which downstream pathway of EGFR is responsible for 14-3-3σ-mediated anoikis resistance in HCC cells. Consequently, the major downstream pathways of EGFR, including AKT, JNK, p38 and ERK1/2 were examined in shNC and sh14-3-3σ cells after EGF stimulation. The phosphorylation level of ERK1/2 was markedly reduced in 14-3-3σ-knockdown HLF and Huh7 cells after EGF stimulation, whereas the total ERK1/2 levels were not affected (Figure 6A and supplementary Figure S7A). Interestingly, the AKT, JNK, and p38 signaling pathways exhibited no such changes between the 14-3-3σ knockdown cells and control cells (Figure S7B). Moreover, ERK1/2 activation was also significantly increased in the 14-3-3σ overexpressing HepG2 cells compared with the control vector after EGF stimulation (Figure 6B). The ERK/MAPK pathway was reported to play a critical role in anoikis resistance in metastatic HCC cells 8, and it is possible that 14-3-3σ induces anoikis resistance in hepatoma cells by regulating ERK1/2 activation. Next, we examined the phosphorylation levels of ERK1/2 after 48 h of cell growth in suspension. As shown in Figure 6C and supplementary Figure S7C, 14-3-3σ silencing in both HLF and Huh7 cells resulted in significantly decreased phosphorylation of ERK1/2 compared with the negative control. Furthermore, the 14-3-3σ overexpressing HCC cells showed increased levels of phosphorylated ERK1/2 compared to the vector control cells (Figure 6D). We also examined the apoptosis-related downstream substrates of ERK1/2 to clarify the molecular mechanisms underlying 14-3-3σ-dependent enhancement of cellular anoikis resistance. The expression of the anti-apoptotic protein Bcl2 was markedly decreased and that of pro-apoptotic Bim was markedly increased in the 14-3-3σ-knockdown HLF and Huh7 cells compared with the shNC cells when grown in suspension (Figure 6E and supplementary Figure S7D). By contrast, overexpression of 14-3-3σ resulted in decreased levels of Bim, and increased levels of Bcl2 in suspension cells (Figure 6F). These results demonstrated that 14-3-3σ had a strong effect on the activation of ERK1/2 and its downstream effectors in response to anoikis. Next, we tested whether 14-3-3σ affects anoikis resistance via the ERK1/2 pathway. We utilized loss-of-function strategies with specific pharmacological inhibitors to block ERK1/2 activities in HCC cells. As shown in Figure 6G, we observed that the ERK1/2 inhibitor U0126 significantly suppressed the activation of ERK1/2. Furthermore, Annexin V/PI staining (Figure 6H-I), the trypan blue exclusion cell viability test(Figure 6J), and caspase 3 activity assay (Figure 6K) showed that the decrease of apoptosis in HepG2 HCC cells under suspension conditions due to 14-3-3σ overexpression was reversed by the ERK1/2 inhibitor U0126. The protein levels of cleaved caspase-3, cleaved PARP, Bcl2 and Bim were also analyzed by western blotting. As shown in Figure 6L, the U0126 inhibitor markedly decreased the protein level of Bcl2, and increased the levels of cleaved caspase-3, cleaved PARP and Bim in 14-3-3σ-overexpressing HepG2 HCC cells in suspension. Moreover, 14-3-3σ reconstitution restored ERK1/2 activation and rescued the anoikis resistance of 14-3-3σ-knockdown HLF cells (Figures 6M-O). Taken together, these findings demonstrate that ERK1/2 is an essential downstream signaling pathway for 14-3-3σ-induced anoikis resistance in HCC cells.

### The EGFR pathway is involved in the enhancement of ERK1/2 activation by 14-3-3σ and anoikis resistance

**Figure 7:**
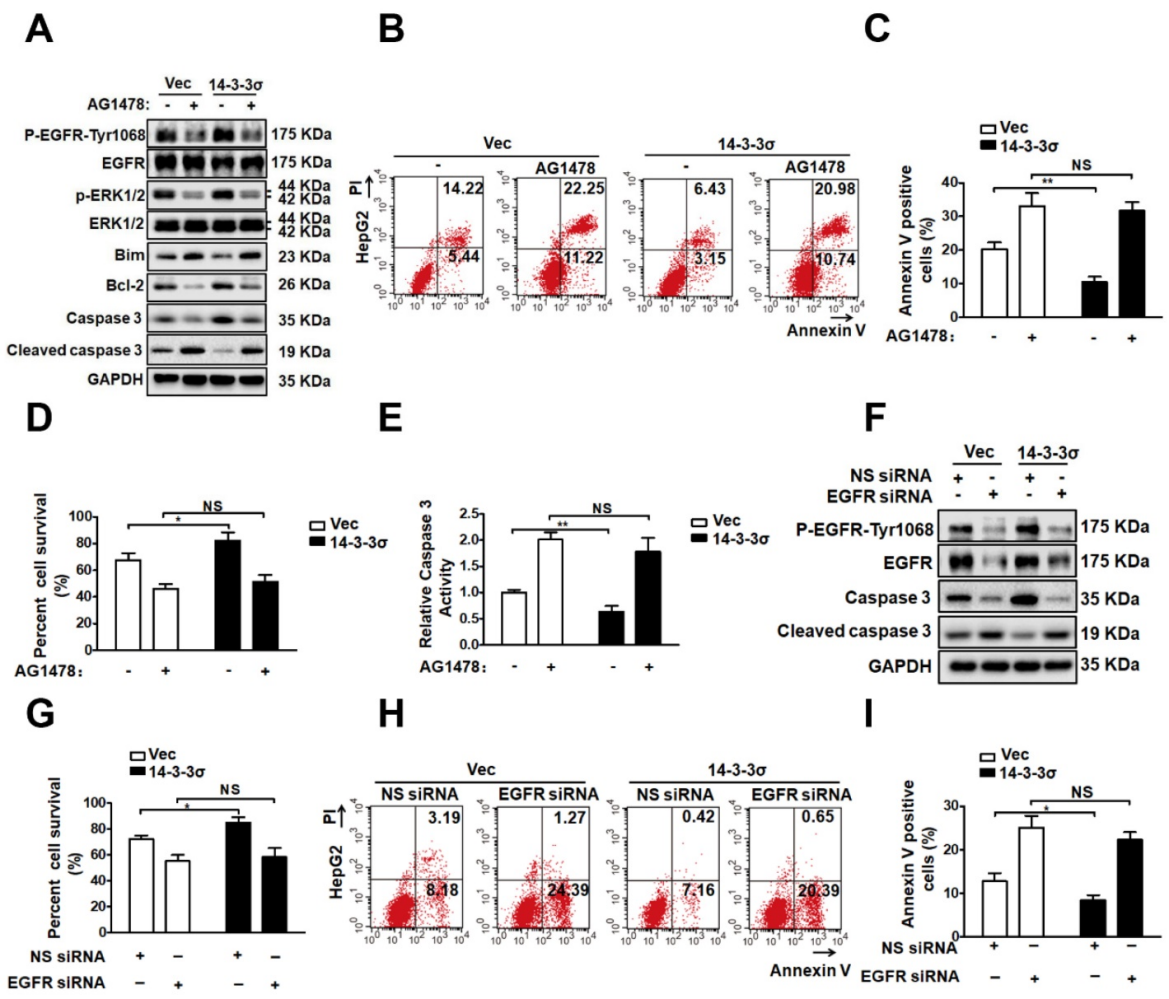
** EGFR pathway is involved 14-3-3σ-enhanced ERK1/2 activation and anoikis resistance. (A-E)** HepG2-Vec and HepG2-14-3-3σ cells were treated with EGFR inhibitor AG1478 (10 μM) and cultured in the detached state for 48 hours. **A,** The expressions of EGFR, p-EGFR-1068, ERK1/2, p-ERK1/2, Bim, Bcl2, Caspase 3, and cleaved caspase 3 were assessed by western blot.** B and C,** Cell apoptosis was assessed with flow cytometric analysis using Annexin V kit. **D**, Cell viability was assessed by Trypan blue exclusion assay.** E,** cell apoptosis was assessed by caspase 3 activity assay. (**F-I**) 32 hours after transfected with 100nM siRNA of EGFR (EGFR siRNA) or negative control (NS siRNA), the HepG2-Vec and HepG2-14-3-3σ cells were cultured in suspension conditions for 48 h.** F,** The expressions of EGFR, p-EGFR-1068, Caspase 3, and cleaved caspase 3 were assessed by western blot. **G,** Cell viability was assessed by Typan blue exclusion assay. **H and I,** Cell apoptosis was assessed with flow cytometric analysis using Annexin V kit.

**Figure 8:**
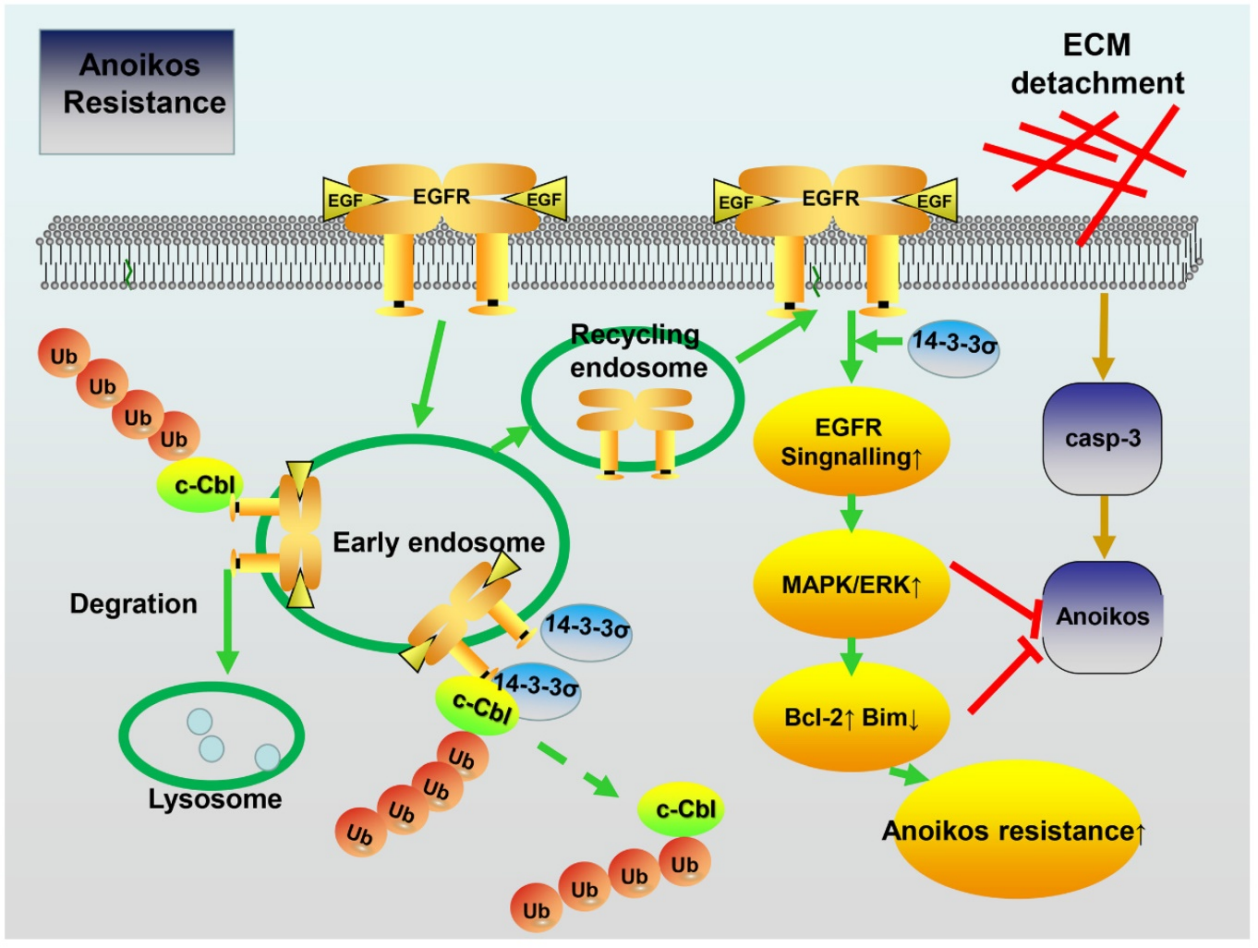
** Schematic model depicting how 14-3-3σ performs anoikis resistance effects in HCC.** 14-3-3σ stabilizes the EGFR protein and positively regulates EGFR signaling by blocking the c-Cbl-EGFR interaction and subsequent c-Cbl-mediated EGFR ubiquitination.

To further clarify the possible role of EGFR in the mechanism through which 14-3-3σ modulates ERK1/2 activation and anoikis resistance, we utilized loss-of-function strategies using the specific inhibitor AG1478 to block EGFR signaling in detached cells. As shown in Figure 7A, treatment with AG1478 completely abrogated the effects of 14-3-3σ on both EGFR and ERK1/2 phosphorylation after 48 h of growth in suspension. Next, experiments were performed to investigate the role of EGFR in ERK1/2 activation and anoikis resistance conferred by 14-3-3σ expression. As shown in Figures 7B and C, EGFR inhibition decreased the anoikis resistance of 14-3-3σ-overexpressing cells, and displayed and increased the population of Annexin V-positive cells in suspension. Moreover, the cell viability assay (Figure 7D), caspase 3/7 activity assay (Figure 7E), and western blot analysis of cleaved caspase 3 (Figure 7A) in HepG2 suspension cells showed that 14-3-3σ-induced anoikis resistance was also reversed by blocking EGFR activity using AG1478. Furthermore, western blot analysis of apoptosis-related proteins showed that the upregulation of Blc2 and downregulation of Bim after detachment in 14-3-3σ-expressing HepG2 cells was reversed by the EGFR inhibitor treatment (Figure 7A). In addition, western blot analysis of cleaved caspase 3 (Figure 7F), cell viability assay (Figure 7G), and Annexin V/PI staining (Figure 7H-I) showed similar results, indicating that the enhanced anoikis resistance of 14-3-3σ-expressing HepG2 cells was blunted by siRNA-mediated EGFR knockdown. To further examine the relationship between 14-3-3σ and EGFR in human cancers, we analyzed the levels of 14-3-3σ and EGFR by IHC in serial sections of HCC samples (Figure S8A). The average score for EGFR was found to be significantly higher in the high 14-3-3σ expression group than in the low expression group (Figure S8B). Moreover, our results indicate that EGFR expression is positively correlated with 14-3-3σ expression in HCC samples (Figure S8C). Taken together, these data demonstrate that 14-3-3σ promotes the anoikis resistance of HCC cells by inhibiting EGFR degradation and thereby activating the EGFR-dependent ERK1/2 signaling pathway (Figure 8).

## Discussion

The roles of 14-3-3σ protein have been studied in many primary tumors and tumor cell lines 11, 12, 15. However, there are few reports on the relationship between 14-3-σ function and HCC. Here, we found that 14-3-3σ was significantly upregulated in HCC tissue samples and related to the prognosis of HCC patients. Anoikis resistance is considered as one of the major mechanisms contributing to metastasis and tumor progression. It is thus important to identify key regulators of anoikis and the mechanisms by which their dysregulation confers anoikis resistance in cancer cells. The 14-3-3σ protein has been implicated in the development of chemo- and radiation resistance of multiple human cancers by regulating DNA repair and inhibiting apoptosis 24, 44. Furthermore, functional proteomic analysis demonstrated that 14-3-3σ contributes to resistance to both mitoxantrone and doxorubicin in MCF7/AdVp3000 cells 26. Moreover, previous studies indicated that 14-3-3σ overexpression might be a mechanism for anoikis resistance in CCA cells 16, 27. Here, we focused on the function of 14-3-3σ in HCC and identified a novel crucial role of 14-3-3σ in anoikis resistance of HCC cells. The results of this study indicate that 14-3-3σ overexpression in human HCC cells significantly increases anoikis resistance. Furthermore, we used a well-established nude mouse model of lung metastasis to confirm that knocking down 14-3-3σ could decrease the formation of lung metastatic foci.

The molecular mechanisms of anoikis resistance in cancer cells are complex, and include overexpression of growth factor receptors, upregulation of integrin signaling, as well as the activation of pro-survival signaling 8. EGFR, a member of the ERBB receptor tyrosine kinase superfamily, seems to be a key factor for anoikis resistance in human cancer cells 32, 45. Various proteins were reported to control the malignant phenotypes of cancerous cells by interacting with EGFR to modulate its signaling pathway 46, 47. Previous studies suggest that 14-3-3σ is also an interaction partner of the EGFR protein complex 33. Accordingly, tumor cells overexpressing 14-3-3σ were found to exhibit increased survival in the presence of cisplatin, attenuated activation of proapoptotic pathways, and enhanced invasiveness in response to EGF 12. Our results indicate that modulation of the EGFR pathway is the key mechanism by which 14-3-3σ promotes anoikis resistance in HCC cells. We showed that 14-3-3σ interacts with EGFR in HCC cell lines, whereby knockdown of 14-3-3σ resulted in significant repression of the EGFR activation in both attached and detached cells. In addition, inactivation of EGFR using its specific inhibitor AG1478 abrogated the 14-3-3σ-mediated resistance to anoikis in HCC cells (Figure 7), thus further supporting the notion that 14-3-3σ confers anoikis resistance by activating the EGFR signaling pathway. Importantly, our mechanistic study demonstrated that knockdown of 14-3-3σ dramatically reduced EGFR levels on the cell surface by accelerating EGF-induced internalization and degradation of EGFR. The EGFR ubiquitination assay showed that knockdown of 14-3-3σ led to an increase of EGF-induced EGFR ubiquitination, while overexpression of 14-3-3σ resulted in inhibition of EGFR ubiquitination. Furthermore, we demonstrated that 14-3-3σ inhibited EGF-induced EGFR degradation after EGFR activation by interfering with the EGFR-c-Cbl interaction and subsequent c-Cbl-mediated EGFR ubiquitination. Finally, our data indicated that competition between 14-3-3σ and c-Cbl for binding to EGFR may constitute a novel mechanism that protects activated EGFR from ubiquitination and degradation. These aspects of the mechanism remain to be explored further. Taken together, the data suggest that 14-3-3σ expression in tumor cells might promote survival by regulating EGFR ubiquitination and sustaining EGFR signaling at the cell surface, ultimately enhancing the anoikis resistance of tumor cells.

Anoikis resistance involves dysregulated expression of growth factor receptors or components of their signaling pathways that inhibit cell death pathways and active pro-survival pathways, such as the Ras/ERK, leading to metastasis by inhibiting anoikis 48. The ERK signaling pathways that are the chief mechanisms for controlling cell survival, differentiation, proliferation, and motility in response to extracellular cues, have been implicated as central regulators of anoikis in many reports 8, 49-51. Here, we demonstrated that 14-3-3σ knockdown impaired the phosphorylation of ERK1/2 after EGF stimulation, while overexpression had the opposite effect. After treatment with U0126, a selective inhibitor of ERK1/2, HCC cell lines displayed a higher rate of apoptosis, thus exhibiting reversion to an anoikis-sensitive state, which indicated that enhanced ERK1/2 activity induced by 14-3-3σ overexpression in HCC suspension cells provides protection against anoikis. Previous studies proposed several mechanisms involving ERK signaling in the protection against apoptotic cell death. ERK/MAPK signaling was found to operate downstream of ErbB2 and EGFR to protect cells from anoikis by facilitating the formation of a protein complex containing Bim-EL, LC8, and Beclin-1 50. Other mechanism by which pro-survival factors exploit the ERK1/2 pathway to protect against apoptotic cell death include the upregulation of the antiapoptotic members of the Bcl-2 family, such as Bcl-2, Bcl-XL and Mcl-1, as well as the downregulation of the proapoptotic protein Bad 8, 52. Many central components of the apoptotic machinery are controlled by ERK1/2 effector pathways. Our results indicate that expression of Bcl2 was markedly decreased, while that of the pro-apoptotic protein Bim was increased in 14-3-3σ-knockdown HCC cells in suspension. Furthermore, when HCC cells with 14-3-3σ overexpression were exposed to anoikis conditions in the presence or absence of the specific ERK inhibitor U0126, the protein levels of Bcl2 or Bim were normalized and the anoikis resistance effect conferred by 14-3-3σ was blocked due to the inactivation of the ERK pathway (Figure 6). Therefore, our findings suggest that 14-3-3σ knockdown-induced suppression of HCC anoikis resistance is likely mediated by the inhibition of ERK1/2 phosphorylation and the downstream apoptotic machinery. Finally, we found that HCC cells treated with the EGFR inhibitor attenuated the 14-3-3σ-mediated enhancement of ERK1/2 phosphorylation and anoikis resistance, as well as normalizing the protein levels of Bcl2 and Bim (Figure 7). Thus, it is possible that 14-3-3σ regulates the anoikis of tumor cells through the EGFR-dependent ERK signaling pathway. However, there are also other potential mechanisms for 14-3-3σ-mediated anoikis resistance in HCC cells. For example, 14-3-3σ knockdown in ICC cells suppressed the expression of MMP2 and MMP9, which significantly enhanced anoikis 16. The role of 14-3-3σ in resisting apoptosis was previously shown to possibly rely on binding to proapoptotic proteins, such as Bax and Bad, resulting in their inactivation 53, 54. Studies have indicated 14-3-3σ inhibits radiation-induced apoptosis and contributes to radioresistance via enhanced NHEJ repair due to the expression of Chk2 and PARP1 24. Su YW et al. demonstrated that 14-3-3σ regulates FOXO transcription factors, which promote cellular survival by preventing apoptosis 55. The roles of 14-3-3σ in HCC pathogenesis and the anoikis-resistance mechanisms are still largely unknown, and further studies of the role of this protein in anoikis resistance of HCC are required. Nevertheless, our data support our hypothesis that 14-3-3σ mediates EGFR-dependent ERK1/2, which in turn may help HCC cells overcome anoikis.

Even though therapeutic targeting of 14-3-3σ is an underdeveloped area of research, our data and other studies have validated 14-3-3σ overexpression could lead to cancer recurrence, radioresistance, chemoresistance, and anoikis resistance, suggesting 14-3-3σ as a potential target in cancer therapy. Presently, small interfering RNAs, antisense, and peptide inhibitors are used for targeting 14-3-3 under experimental conditions 56. However, the clinical feasibility of using siRNA, anti-sense or peptide inhibitors as therapies is still a challenge that needs to be met 57. Our study suggest that targeting 14-3-3σ or the downstream pathways regulated by 14-3-3σ may sensitize cells to apoptosis and serve as effective anticancer strategies. This is an area of research which needs more focus and may bring the greatest benefit to HCC patients with 14-3-3σ overexpressing tumors.

In summary, we showed that 14-3-3σ was upregulated in HCC tumors compared with adjacent normal tissues, and 14-3-3σ expression was correlated with clinical parameters such as pathological stage, clinical stage, and disease survival rate. Downregulation of 14-3-3σ reduced the anoikis resistance of HCC cells, as well as inhibiting the growth and metastasis of HCC *in vivo*. Although other, unknown mechanisms may contribute to the observations of this study, the anoikis resistance conferred by overexpression of 14-3-3σ is an important mechanism through which 14-3-3σ provides protection for HCC cells. Significantly, the present study uncovered an uncharacterized mechanism by which 14-3-3-σ contributes to anoikis resistance in HCC cells, likely by inhibiting EGFR degradation and thereby activating the EGFR-dependent ERK1/2 pathway. These findings provide new insights into the pathophysiological roles of 14-3-3σ in HCC metastasis and suggest that 14-3-3σ is a potential therapeutic target for the treatment of HCC.

## Supplementary Material

Supplementary figures.

## Figures and Tables

**Table 1:** Correlation of the clinicopathological characteristics with 14-3-3σ expression in HCC.

Clinicopathological features		14-3-3σ expression		
	N	Low	High	*P* Value
Gender*				
Male	78	30	48	1.000
Femanle	7	3	4	
Age*				
≤50	44	17	27	0.932
>50	41	16	25	
HBV*				
Negative	4	3	1	0.294
Positive	81	30	51	
Cirrhosis*				
NO	23	5	18	0.078
YES	62	28	34	
Tumor size*				
≤5	33	17	16	0.070
>5	52	16	36	
Tumor encapsulation*				
Complete	49	20	29	0.822
None	36	13	23	
Tumor number*				
Solitary	69	28	41	0.578
Multiple(≥2)	16	5	11	
TNM clinical stage *				
I-II	60	28	32	0.028
III-IV	25	5	20	
Vascular invasion*				
NO	52	27	25	0.003
YES	33	6	27	
Differentiation*				
I-II	37	13	24	0.655
III-IV	48	20	28	
BCLC stage*				
A	41	21	20	0.023
B+C	44	12	32	
				

Abbreviations: TNM, tumor node metastasis; BCLC: Barcelona Clinic Liver Cancer. Bold text represents *P*-values with significant difference.

## Abbreviations

HCC: hepatocellular carcinoma
EGFR: epidermal growth factor receptor
c-Cbl: casitas B lineage lymphoma
ERK1/2: extracellular signal-regulated kinase 1/2
MAPK: mitogen-activated protein kinases
ECM: extracellular matrix
TNM: tumor node metastasis
BCLC: Barcelona Clinic Liver Cancer
PARP: poly (ADP-ribose) polymerase
PI: propidium iodide
IP: immunoprecipitation
FACS: fluorescence-activated cell sorting
Ub: ubiquitination
E3: ubiquitin ligases
Bcl2: B-cell lymphoma 2
Bim: B-cell lymphoma 2 interacting mediator of cell death
Bcl-XL: B-cell lymphoma- extra-large
