# Supplementary material for: The 14-3-3σ protein promotes HCC anoikis resistance by inhibiting EGFR degradation and thereby activating the EGFR-dependent ERK1/2 signaling pathway
Source: Theranostics. 2021 Jan 1;11(3):996–1015. doi: 10.7150/thno.51646 (PMC7738881; doi:10.7150/thno.51646)
Supplement: Supplementary file 1 — Supplementary figures. [file thnov11p0996s1.pdf]

Supplementary material:

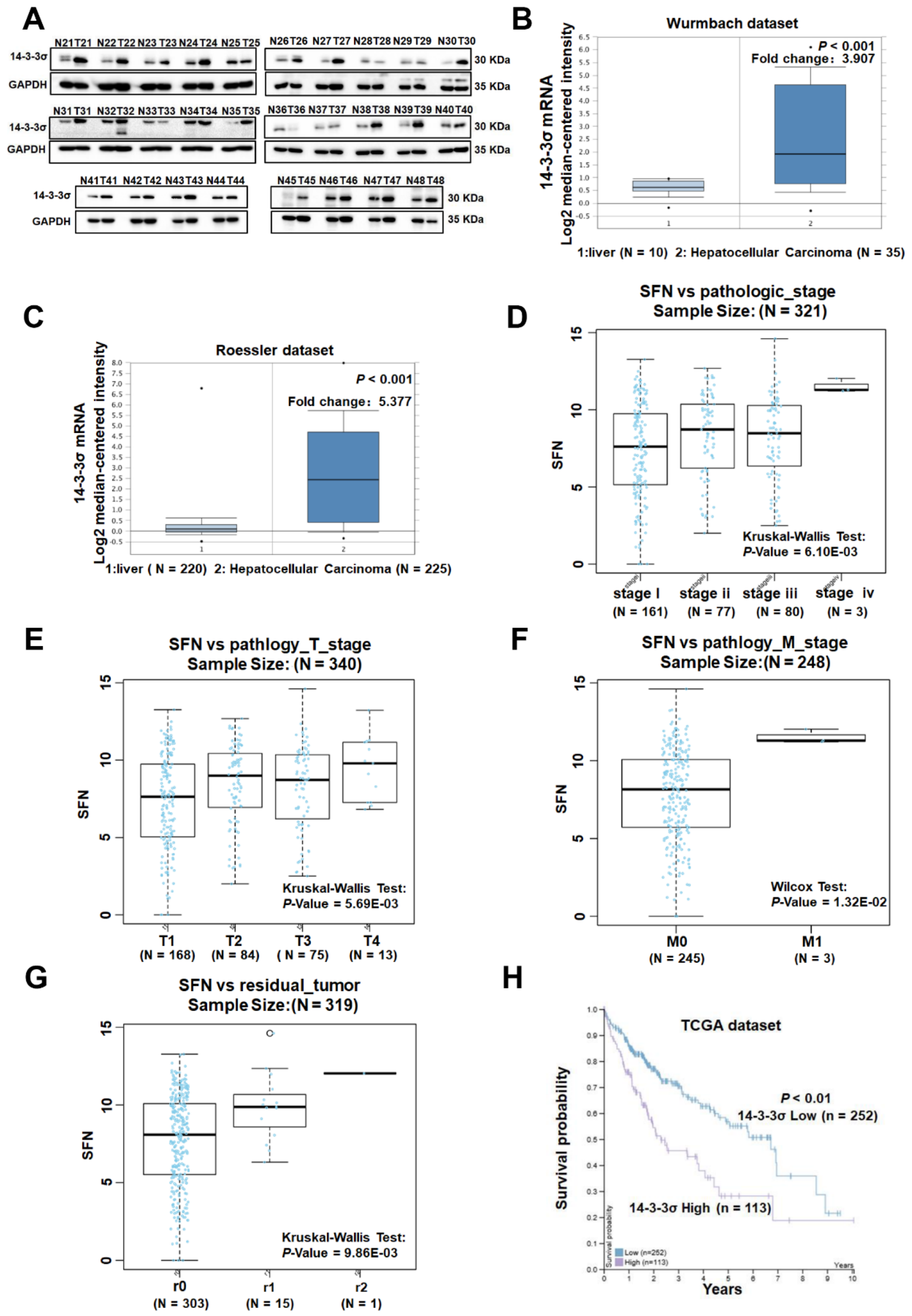

Figure S1. 14-3-3σ protein is significantly upregulated in HCC tissues and correlated with HCC patient survival. (A) Expression level of 14-3-3σ in 48 paired

HCCs with their corresponding non-cancerous tissues. **(B** and **C)** 14-3-3σ mRNA levels in normal liver and hepatocellular carcinoma in the Dataset analysed by Oncomine. Data were collected from Oncomine database (https://www.oncomine.org). (**D-G**) In the Cancer Genome Atlas (TCGA) database, 14-3-3σ mRNA levels in different clinical stage (stage I-IV), pathology T stage (T Stages 1-4), pathology M stage (M stage 0-1), and residual tumor (r 0-2) HCC tissues were revealed by TCGA data-mining. Data were analysed using the Kruskal–Wallis test or Wilcox Test. **(H)** Kaplan-Meier analyses of the correlations between 14-3-3σ mRNA levels and overall survival of 365 patients with HCC. Data were collected and analyzed at the website (http://www.cbioportal.org); the original data can be retrieved from The Cancer Genome Atlas (TCGA) database.

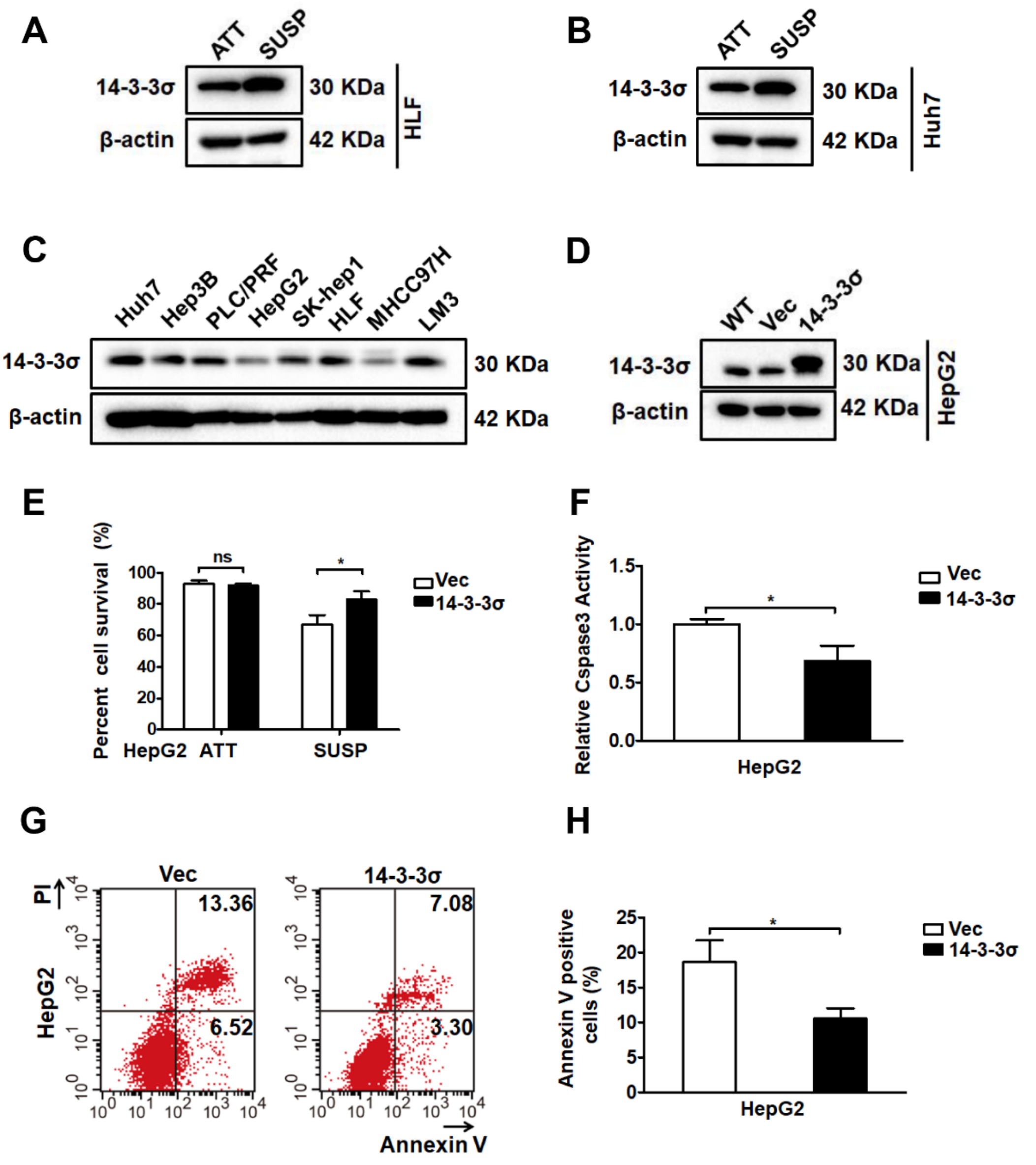

Figure S2. 14-3-3σ enhances anoikis resistance in hepatocellular carcinoma cells. **(A and B)** HCC HLF and Huh7 cells were grown in attached (ATT) or suspension (SUSP) conditions for 48h followed by cell lysate preparation. The expression levels of 14-3-3σ were determined by immunoblotting. **(C)** 14-3-3σ expression in several HCC cell lines was examined using western blot analysis. **(D)** Relatively high expression of 14-3-3σ was confirmed by western blotting in the 14-3-3σ-overexpressing HCC HepG2 cells compared with the vector control cells. **(E-H)** The in vitro anoikis assay indicated that overexpression of 14-3-3σ significantly decreased the anoikis rate of HepG2 cells when cells are suspended for

48 h, as characterized by Typan Blue Staining Cell Viability Assay (**E**), Caspase 3 activity assay (**F**), and Annexin V/PI staining **(G** and **H)**. Data are shown as mean ± SD. **P* < 0.05, by Student's t test.

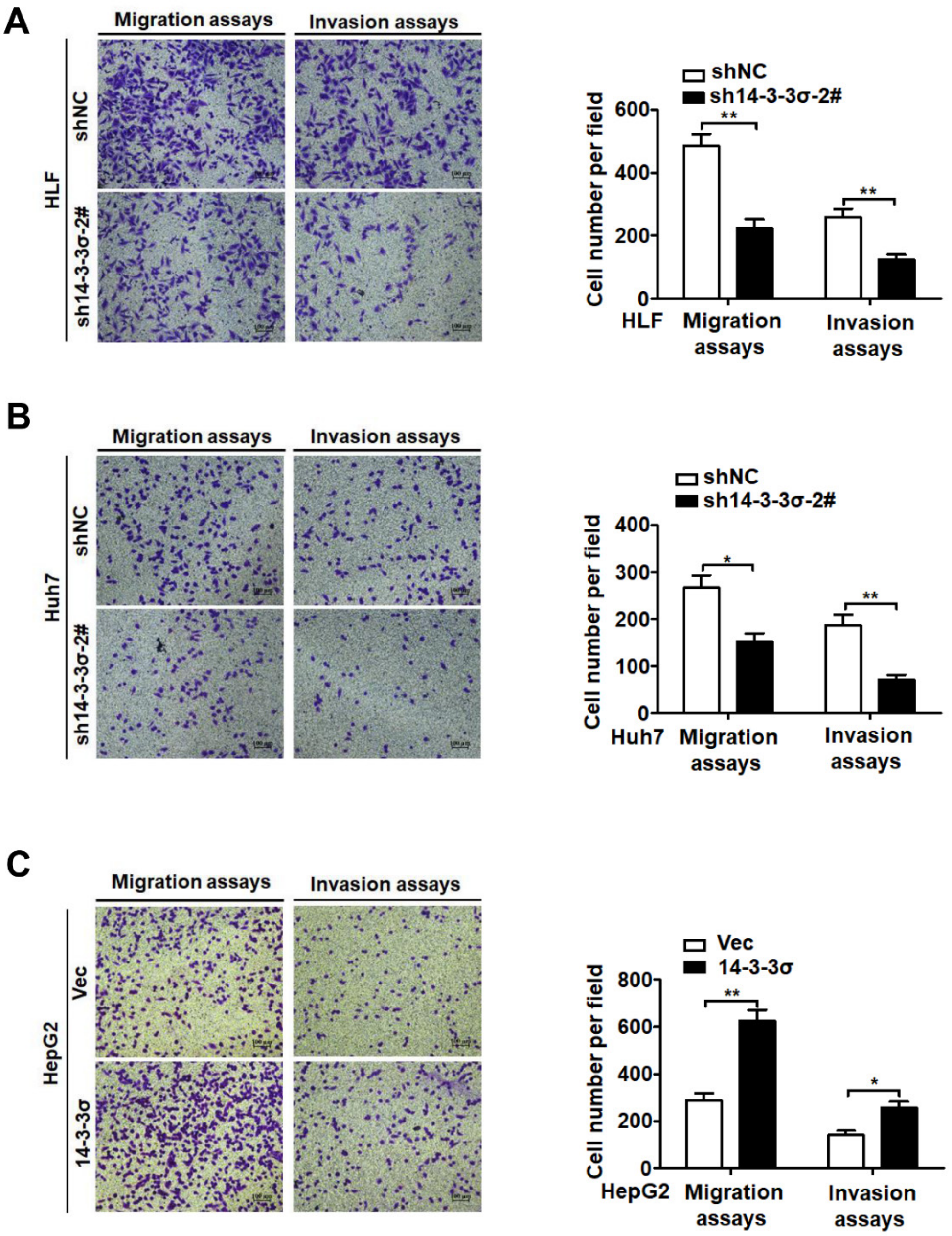

Figure S3. 14-3-3σ enhances the migration and invasion of HCC cells *in vitro.* (**A** and **B**) Migration and invasion assays were used to test the migration and invasion abilities of 14-3-3σ-knockdown HLF and Huh7 cells. (C) The migration and invasion abilities of HCC cell were determined by transwell assays in 14-3-3σ-overexpressing

HepG2 cells. The data are represented as the mean ± SD, n=3. *P < 0.05; **P < 0.01.

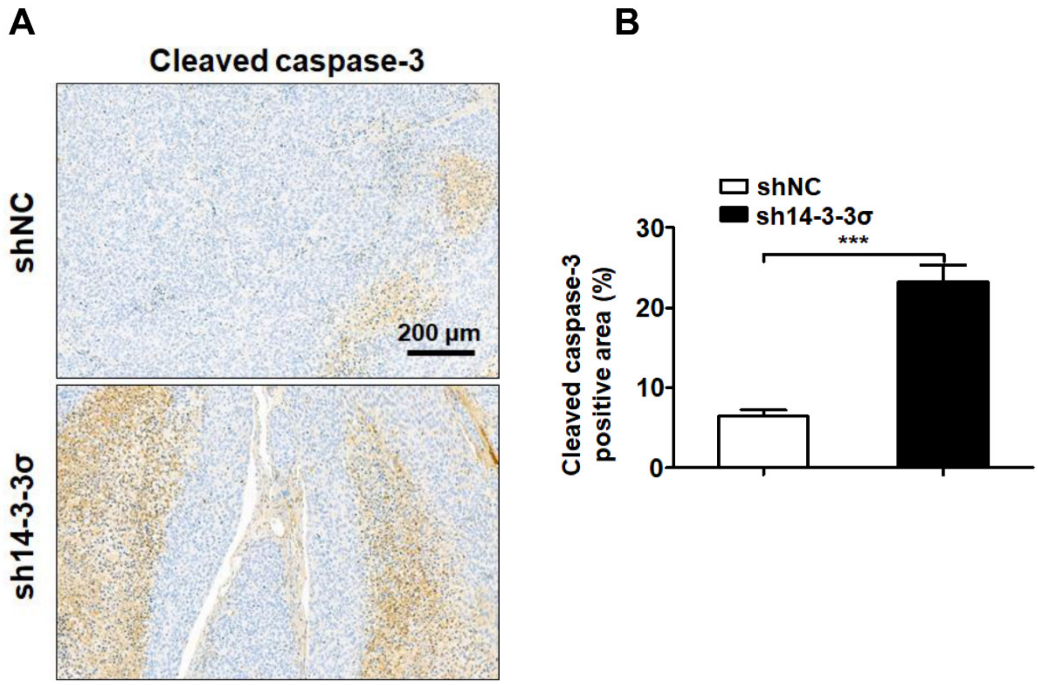

Figure S4. 14-3-3σ knockdown enhanced HCC cell apoptosis in intrahepatic tumor implantation models. (**A**) Representative images of IHC staining of cleaved caspase-3 in tumors (n = 6). Scale bar: 200 μm. (**B**) Quantitative analysis of cleaved caspase-3 staining in intrahepatic tumor implantation models. The data are represented as the mean ± SD, n = 6, ***P < 0.001.

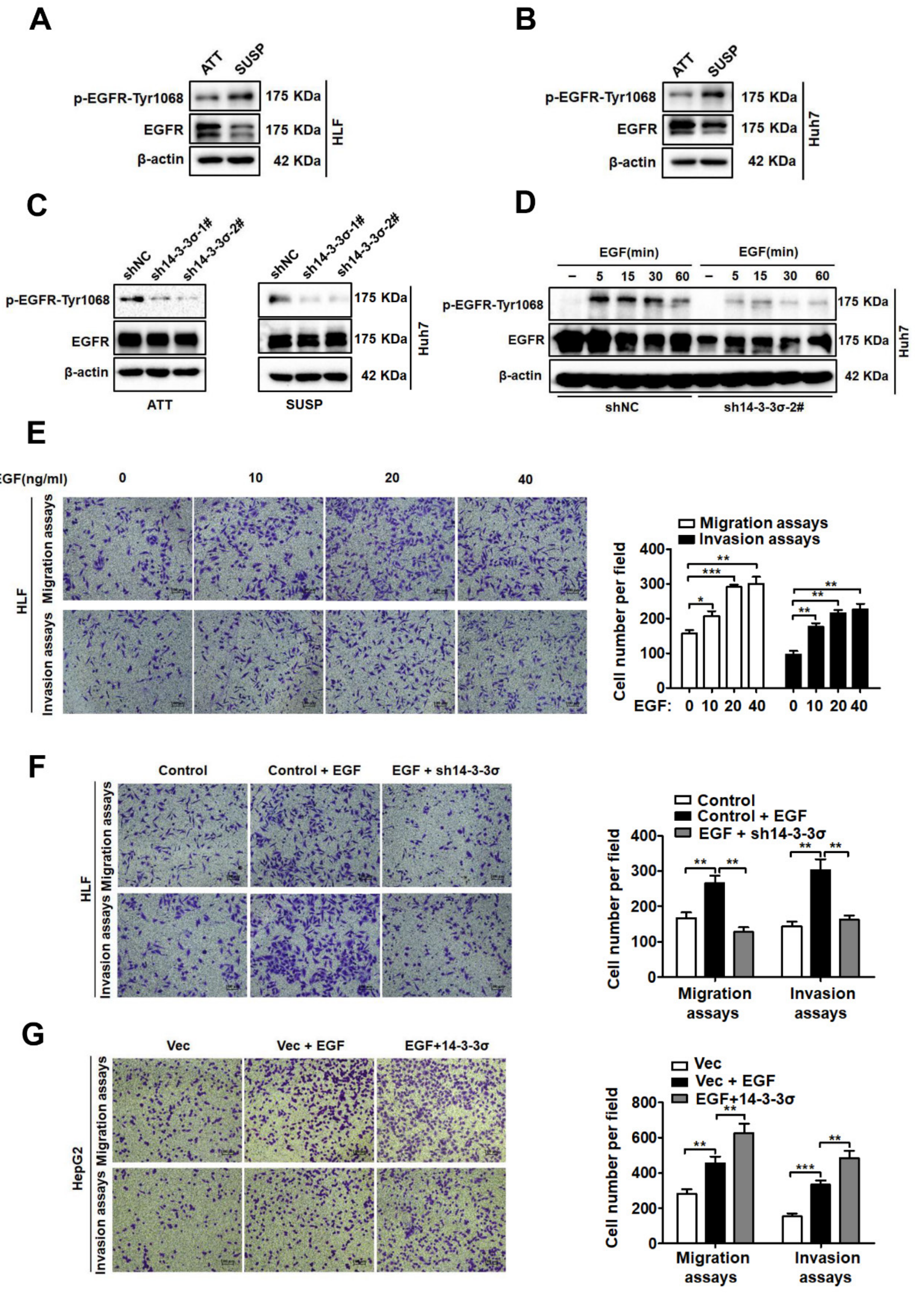

Figure S5. 14-3-3σ could modulates EGFR protein levels during EGF treatment. (A and B) HCC HLF and Huh7 cells were grown in attached (ATT) or suspension (SUSP) conditions for 48h followed by cell lysate preparation. The total expression levels and phosphorylation levels of EGFR (p-EGFR-Tyr1068) were determined by

immunoblotting. **(C)** Huh7 shNC or sh14-3-3σ cells lines were cultured as attached monolayers or in suspension (SUSP) conditions for 48 h. Cells were harvested and subject to western blot analysis using the indicated antibodies. **(D)** Huh7 shNC and sh14-3-3σ cells were serum starved overnight and treated with 80 ng/ml EGF for the indicated time period. Whole-cell lysates were prepared and subject to western blot analysis using the indicated antibodies. **(E)** HCC HLF cells were treated with different concentrations of EGF for 12 h and underwent transwell assays to examine the effect of EGF on the migration and invasion of the HCC cells. **(F-G)** Effect of 14-3-3σ on EGF-stimulated cell migration and invasion. **F**, 14-3-3σ knockdown (sh14-3-3σ) and control HCC HLF cells were incubated with 40 ng/mL EGF for 12 h and the cell migration/ invasion rate was determined by transwell assays. **G**, HCC hepG2 cells expressing empty vector or 14-3-3σ were incubated with 40 ng/mL EGF for 12 h and the cell migration/invasion rate was determined by transwell assays. The data are represented as the mean ± SD, n=3. *$P < 0.05$; **$P < 0.01$; ***$P < 0.001$.

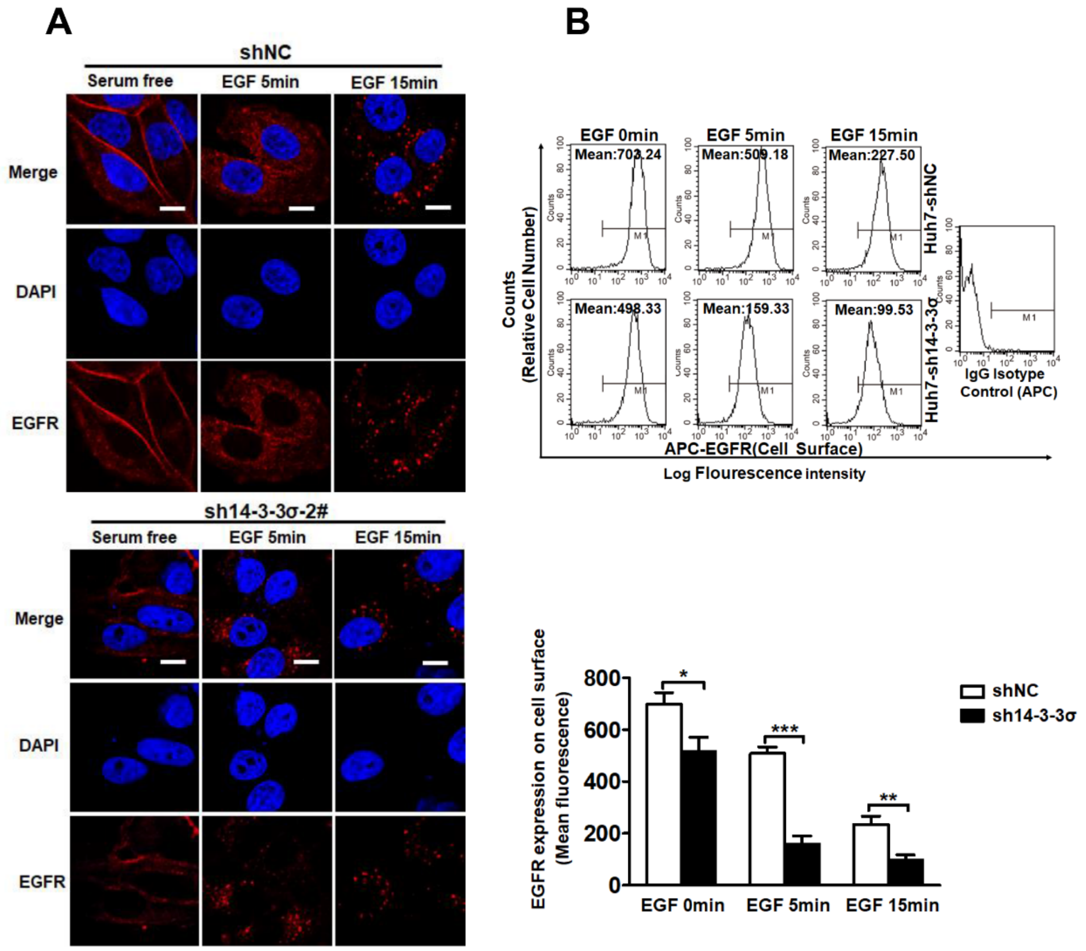

Figure S6. 14-3-3σ inhibited loss of surface EGFR signal following EGF-induced EGFR degradation. (**A**) Huh7 shNC and sh14-3-3σ cells were serum starved overnight and treated with 40 ng/ml EGF for the indicated times. Cells were fixed and analyzed by immunofluorescence confocal microscopy using an antibody against EGFR (red). Nuclei were stained with DAPI (blue). Scale bar, 10 μm. (**B**) The cell surface expression levels EGFR in HCC Huh7 shNC and sh14-3-3σ cells were determined using flow cytometry. The data are presented as the mean ± SD; *$P < 0.05$; **$P < 0.01$; ***$P < 0.001$.

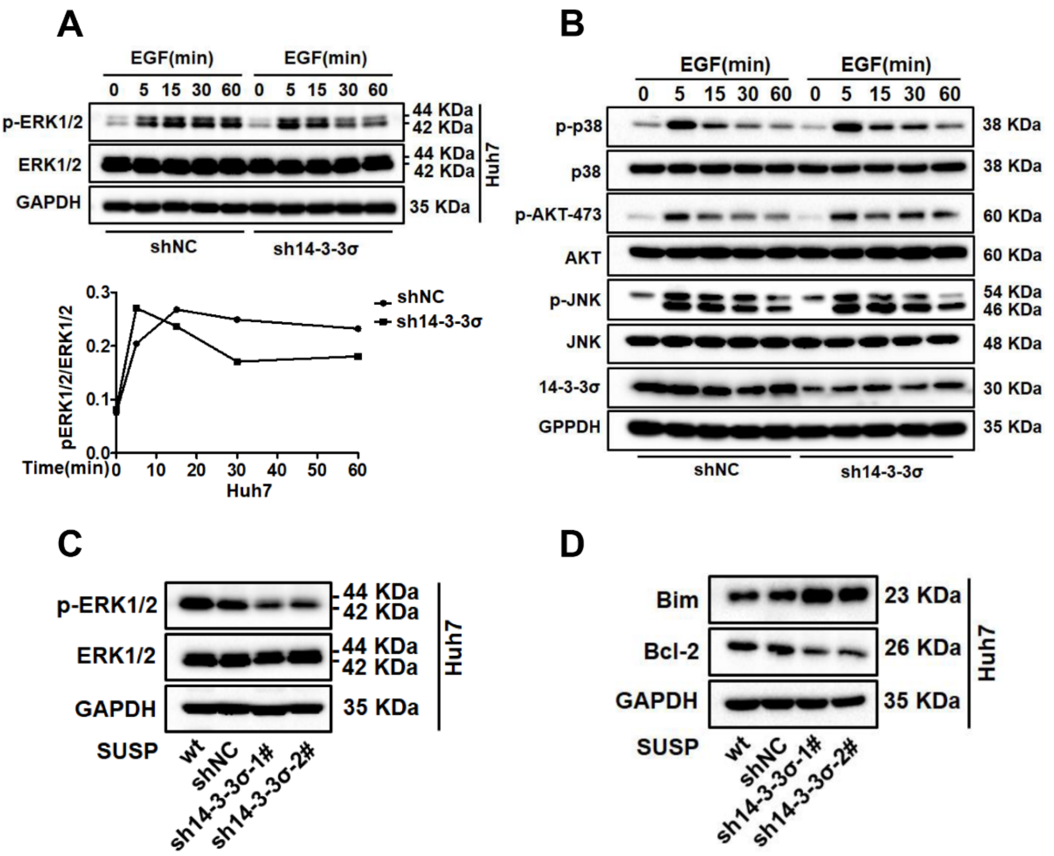

Figure S7. 14-3-3σ confers anoikis resistance via the activation of the ERK 1/2 pathway. (A) shNC or sh14-3-3σ Huh7 cells were starved with serum-free medium for 12 h, then added EGF (40 ng/ml) and collected at the indicated time points. The levels of ERK1/2 and phosphorylated ERK1/2 were analyzed by western blot. **(B)** shNC or sh14-3-3σ HLF cells were treated with EGF (40 ng/mL) for the indicated intervals. Cell lysates were subjected to western blotting with the indicated antibodies referred to the main downstream signals. **(C and D)** The 14-3-3σ-knockdown Huh7 cells were grown under suspension conditions. Cell lysates were prepared 48 h after suspension culture and immunoblotted with the indicated antibodies.

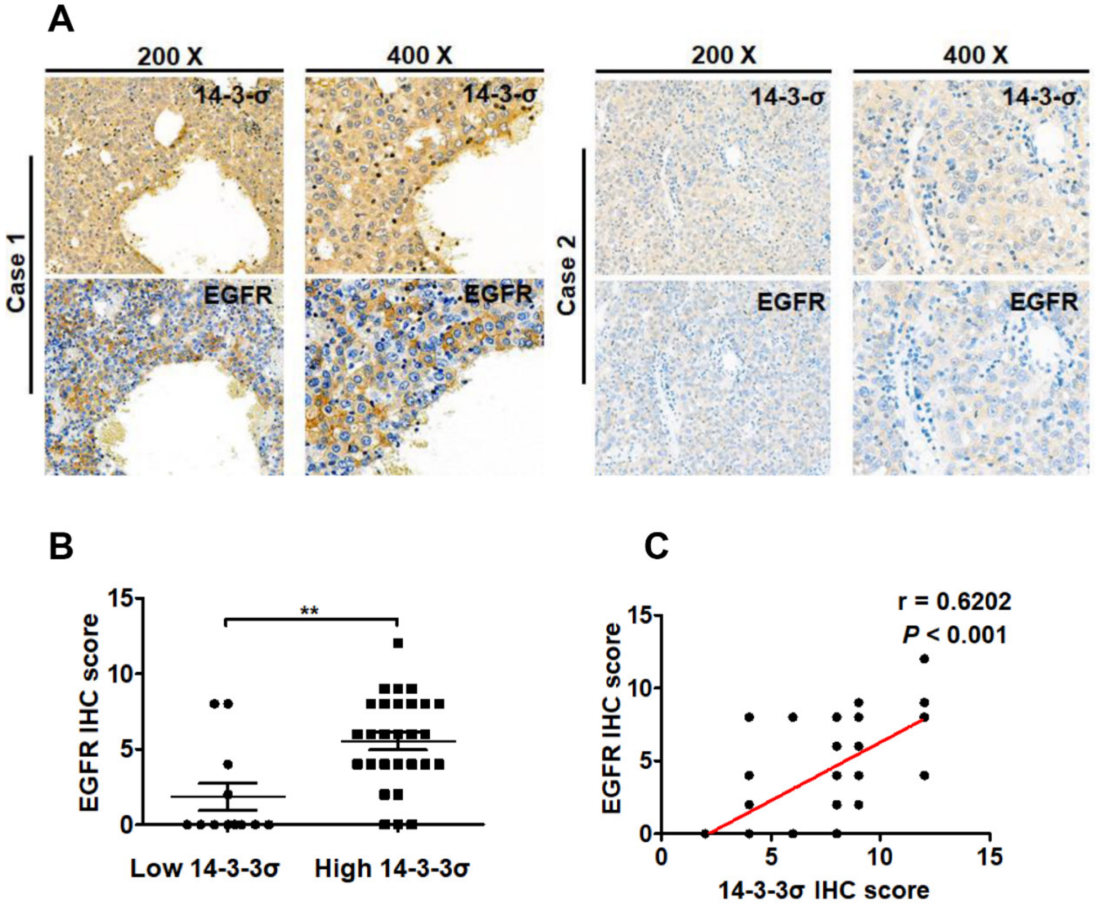

Figure S8. Expression of 14-3-3σ and EGFR are positively correlated in human HCC tissues. (A) Serial sections of HCC tissues were subjected to IHC staining with antibodies against 14-3-3σ and EGFR. In case 1, high expression of 14-3-3σ in HCC cancer tissues was accompanied by elevated EGFR. In case 2, low expression of 14-3-3σ was accompanied by the absence of EGFR. **(B)** Distribution of EGFR expression level in high 14-3-3σ expression level group and low 14-3-3σ expression level group tissues. **(C)** Spearman correlation analysis between 14-3-3σ and EGFR expression. N = 40; *$P < 0.05$; **$P < 0.01$.
